# Association between Changes in Muscle Quality with Exercise Training and Changes in Cardiorespiratory Fitness Measures in Individuals with Type 2 Diabetes Mellitus: Results from the HART-D Study

**DOI:** 10.1371/journal.pone.0135057

**Published:** 2015-08-07

**Authors:** Martin Sénéchal, Neil M. Johannsen, Damon L. Swift, Conrad P. Earnest, Carl J. Lavie, Steven N. Blair, Timothy S. Church

**Affiliations:** 1 Faculty of Kinesiology, Fredericton, New Brunswick, Canada; 2 University of New Brunswick, Fredericton, New Brunswick, Canada; 3 Department of Preventive Medicine, Pennington Biomedical Research Center, Baton Rouge, Louisiana, United States of America; 4 The Louisiana State University, Baton Rouge, Louisiana, United States of America; 5 Department of Kinesiology, East Carolina University, Greenville, North Carolina, United States of America; 6 Center for Health Disparities, East Carolina University, Greenville, North Carolina, United States of America; 7 John Ochsner Heart and Vascular Institute, Ochsner Clinical School-The University of Queensland School of Medicine, New Orleans, Louisiana, United States of America; 8 University of South Carolina, Columbia, South Carolina, United States of America; Texas Tech University Health Science Centers, UNITED STATES

## Abstract

**Introduction:**

Type 2 diabetes mellitus (T2DM) is associated with a reduction in muscle quality. However, there is inadequate empirical evidence to determine whether changes in muscle quality following exercise are associated with improvement in cardiorespiratory fitness (CRF) in individuals with T2DM. The objective of this study was to investigate the association between change in muscle quality following a 9-month intervention of aerobic training (AT), resistance training (RT) or a combination of both (ATRT) and cardiorespiratory fitness (CRF) in individuals with T2DM.

**Material and Methods:**

A total of 196 participants were randomly assigned to a control, AT, RT, or combined ATRT for a 9-months intervention. The exposure variable was change in muscle quality [(Post: leg muscle strength/leg muscle mass)-[(Pre: leg muscle strength/leg muscle mass)]. Dependent variables were change in CRF measures including absolute and relative VO_2peak_, and treadmill time to exhaustion (TTE) and estimated metabolic equivalent task (METs).

**Results:**

Continuous change in muscle quality was independently associated with change in absolute (β = 0.015; p = 0.019) and relative (β = 0.200; p = 0.005) VO_2peak_, and TTE (β = 0.170; p = 0.043), but not with estimated METs (p > 0.05). A significant trend was observed across tertiles of change in muscle quality for changes in absolute (β = 0.050; p = 0.005) and relative (β = 0.624; p = 0.002) VO_2peak_ following 9 months of exercise training. No such association was observed for change in TTE and estimated METs (p > 0.05).

**Discussion:**

The results from this ancillary study suggest that change in muscle quality following exercise training is associated with a greater improvement in CRF in individuals with T2DM. Given the effect RT has on increasing muscle quality, especially as part of a recommended training program (ATRT), individuals with T2DM should incorporate RT into their AT regimens to optimize CRF improvement.

**Trial Registration:**

Clinicaltrials.gov NCT00458133

## Introduction

Type 2 diabetes mellitus is characterized by an alteration of peripheral glucose metabolism[1]. Muscle mass is the major peripheral site of glucose uptake, where ~85% of total glucose uptake occurs[2]. Paradoxically, individuals with type 2 diabetes mellitus present with higher muscle mass[3]. However, type 2 diabetes mellitus patients are muscularly weaker[4] compared to normoglycemic individuals, suggesting a significant reduction in muscle quality (muscle quality: muscle strength per region of lean body mass)[4–6]. Prospective data from the Health, Aging, and Body Composition study showed an accelerated reduction of muscle quality in individuals with type 2 diabetes mellitus[5, 7, 8]. This is clinically important, as reduced muscle quality is negatively associated with physical function[4], quality of life[5], metabolic risk factors[5, 9], and higher mortality risk (13–30%)[10, 11]. Therefore, understanding and identifying behaviors associated with improved muscle quality may contribute to the development of effective interventions for individuals with type 2 diabetes mellitus.

Cardiorespiratory fitness (CRF) is a modifiable risk factor that protects against premature mortality[12]. Regular exercise enhances CRF[13, 14], and high CRF attenuates premature mortality associated with cardiovascular disease (CVD)[15] and type 2 diabetes mellitus[16]. Prospective data showed a steep inverse association between CRF and mortality in adults with type 2 diabetes mellitus[17, 18]. In fact, for each increase of 1 metabolic equivalent task (METs) during an exercise test, a 12% increase in survival was observed[15]. These results suggest that individuals with type 2 diabetes mellitus could benefit from an exercise intervention aimed at increasing CRF. Clearly, greater efforts are needed to reduce the burden of CVD associated with type 2 diabetes mellitus.

Exercise training is a cornerstone in the prevention and the management of type 2 diabetes mellitus. Recent data by our group[19] and others[20, 21] have demonstrated that a combination of aerobic training (AT) and resistance training (RT) effectively increase CRF(6.0% to 10.3%). Similarly, individuals with type 2 diabetes mellitus who perform exercise to increase muscle strength experience improvements in CRF[22]. However, the contribution of an exercise-induced increase in muscle quality to the improvement in CRF remains unclear. More specifically, it is unknown whether a combination of AT and RT would generate the greatest improvement in muscle quality and whether this increase in muscle quality will lead to significant improvement in CRF in individuals with type 2 diabetes mellitus.

In the light of these observations, we performed a sub-analysis from the Health benefits of Aerobic and Resistance Training in individuals with type 2 Diabetes mellitus (HART-D) study. The first objective of this study was to investigate whether an increase in muscle quality following 9 months of exercise training is associated with improved CRF measures, including peak oxygen consumption (VO_2peak_), treadmill time to exhaustion (TTE), and maximal estimated METs. Our second objective was to determine whether individuals performing AT and RT with the greatest change in muscle quality would present the greatest improvement in CRF measures following 9 months of exercise training.

## Materials and Methods

### Participants

Sedentary individuals with type 2 diabetes mellitus aged between 30–75 years old were enrolled in HART-D study. Participants were excluded if they had a body mass index (BMI) of 48.0 kg/m^2^ or higher, blood pressure of 160/100 mmHg or higher, fasting triglycerides of 500 mg/dL or higher, use of insulin pump, urine protein greater than 100 mg/dL, serum creatinine greater than 1.5 mg/dL, history of stroke, advanced neuropathy or retinopathy, or any serious medical condition that prevented participants from adhering to the exercise protocol. The Pennington Biomedical Research Center and the Institutional Review Board approved the protocol annually, and all participants gave written informed consent before starting the trial. The study protocol as well as the supporting CONSORT checklist is available as supporting information (S1 Protocol and S1 CONSORT Checklist).

### Recruitment

Participants in the HART-D study were recruited from the greater Baton Rouge, Louisiana area using media, mailers, and community events between April 2007 and August 2009. Eligible participants underwent a medical examination. A medical review confirmed type 2 diabetes status, while age, sex, race/ethnicity, medications, and duration of type 2 diabetes were collected through a written self-report questionnaire. Medical staff reviewed and verified all the information collected by the study staff.

### Study Design

The methodology for HART-D has been previously described[19]. In brief, after completing run-in and baseline assessments, 262 participants were randomly assigned to control, AT, RT, or a combined AT and RT group (ATRT) for a 9-month intervention. A significant number of participants (~17%) from the control group experienced an increase in HbA_1c_ and the data monitoring and safety board discontinued randomization in the control group resulting in an unequal distribution of participants across the intervention groups.

### Randomization

As described in the main paper[19], randomization sequencing was created by an independent investigator using a computer. Briefly, permuted blocks of equal length with fixed numbers of treatment allotments were used for the randomization.

### Blinding

As for the HART-D study, assessment staff were blinded to the intervention group as they were in a different building. Effort was made to maintain blinding of the research staff from the participants’ intervention groups and participants were reminded not to disclose their intervention assignment. However, the nature of this study prevents blinding exercise staffs.

**Fig 1:**
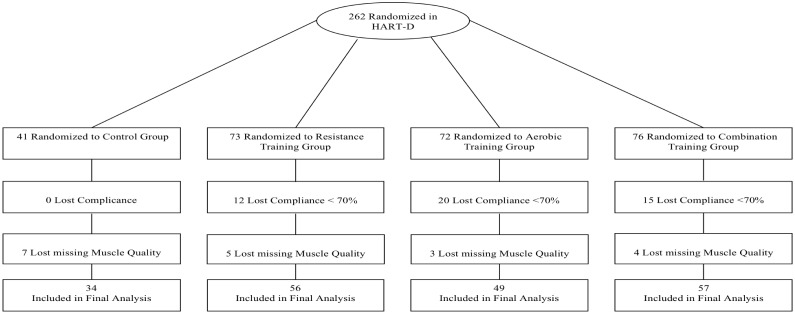
Consort diagram.

The current study is a secondary analysis of the HART-D study. Of the 262 participants randomized in the original study, 47 participants were excluded due to low exercise compliance (<70%) and 19 were excluded because they had missing data for muscle quality index, including eight for DXA and 11 for muscle strength by Biodex. Consequently, 196 participants were included in the present study (Fig 1).

### Exercise Intervention

#### Control

Participants randomized to the non-exercise control group were asked to maintain their current diet and physical activity levels. They were offered weekly stretching and relaxation classes during the 9-month study period.

#### Aerobic Training (AT)

We standardized the exercise prescription to body weight and estimated that 150 minutes per week of moderate intensity exercise was equivalent to 12 kcal/kg body weight per week[19]. Exercise intensity ranged from 50% to 80% of VO_2peak_. Trained staff supervised all exercise sessions and participants were weighed weekly to calculate their prescribed kcal/kg per week. Each session had a 5-minute warm-up and cool-down period. The American College of Sports Medicine equations were used to estimate caloric expenditure rate and time required per session[23].

#### Resistance Training (RT)

Participants exercised 3 days per week with each session consisting of 2 sets of 4 upper body exercises (bench press, seated row, shoulder press, and lat pull down), 3 sets of 3 lower body exercises (leg press, leg extension, and leg curl), and 2 sets of abdominal crunches and back extensions. Each set consisted of 10 to 12 repetitions. When a participant was able to complete 12 repetitions for each set of exercises on 2 consecutive exercise sessions, the prescribed weight was increased. Participants took a rest of about 60–90 seconds between each set as recommended by the American College of Sports Medicine[23].

#### Aerobic and Resistance Training (ATRT)

For the ATRT group, we selected an AT dose of 10 kcal/kg per week and 2 sessions of RT per week. RT consisted of 1 set of 10 to 12 repetitions from 9 exercises previously described. Once the participant was able to complete 12 repetitions for each set of exercises on 2 consecutive exercise sessions, the prescribed weight was increased. We decided on a reduced AT dose and number of sets in the RT group to ensure an equal time commitment between all exercise groups.

### Outcomes and Measurements

#### Anthropometric measures and body composition

Weight was measured on a GSE 450 electronic scale (GSE Scale Systems, Novi, Michigan) and height was measured using a standard stadiometer. Body mass index [BMI: body weight (kg)/height (m^2^)] was then calculated. Waist circumference was measured to the nearest 0.1 cm at the level of the iliac crest while the subject was at minimal expiration. Percent body fat, total and trunk fat mass as well as total lean body mass were measured with Dual-energy x-ray absorptiometry (DXA) scans using the QDR 4500A whole-body scanner (Hologic Inc, Bedford, Massachusetts). After performing whole body scans, a standardized procedure was followed to measure trunk fat mass. Briefly, the top and the bottom horizontal analysis lines were placed just under the jaw and above the iliac crest. The vertical analysis lines are placed on either side of the chest and spine of the whole body. Finally, the points on both shoulders were positioned between the head of the humerus and scapula at the glenoid fossa on the respective shoulder, while the vertical line along the spine was placed closely to the spine and matched for curvature.

#### Hemoglobin A_1c_

Hemoglobin A_1c_ (HbA_1c_) was assessed at baseline and following the intervention. Baseline and post-intervention HbA_1c_ was obtained by venipuncture after a 10-h fast and analyzed with a Beckman Coulter Synchron LX automated analyzer (Fullerton, CA).

#### Cardiorespiratory fitness

CRF was estimated by a VO_2max_ test conducted on a treadmill (Trackmaster 425, Carefusion, Newton, Kansas) with respiratory gases sampled using a True Max 2400 Metabolic Measurement Cart (ParvoMedics, Salt Lake City, Utah). The maximal test was performed using a modified Balke protocol. Briefly, participants self-selected a walking pace and the grade increased by 2% every 2 minutes until exhaustion; VO_2peak_ was reported relative to total body weight. The same speed was used for baseline and post-intervention testing.

#### Treadmill time to exhaustion (TTE)

TTE was determined from the VO_2max_ test performed at baseline and follow-up. The TTE was reported as maximal time in minutes for the overall test duration.

#### Estimated metabolic equivalent task (METs)

Estimated METs were derived from the maximal speed and grade reached during the CRF test at baseline and follow-up using estimated oxygen uptakes from American College of Sports Medicine equations divided by 3.5. We have also quantified CRF by maximal estimated METs as measured VO_2_ is not always available in most cardiology and clinical settings. Therefore, quantification of METs is relevant from a clinical perspective. Additionally, previous epidemiological studies [16, 24], which have observed relationships between CRF and mortality, have typically used estimated METs based on treadmill speed and grade and not measured cardiopulmonary response to quantify CRF. Thus, including estimated METs in the present study will allow us to compare our results with the literature.

#### Muscle quality

A Biodex System 3 dynamometer (Biodex Medical Systems, Shirley, New York) was used to perform muscle strength assessments. Concentric isokinetic knee extension was tested to determine peak torque (60°/s). Participants performed three sets of five maximal repetitions and peak torque was determined as the highest score of the 5 maximal repetitions. All muscle strength assessments were performed with the right leg.

The common belief is that muscle mass significantly explains muscle strength. However, the correlation between muscle mass and muscle strength vary considerably among individuals (r = 0.29 to 0.68)[25, 26]. In addition, individuals with type 2 diabetes mellitus present with higher muscle mass[3] and lower muscle strength[4], which suggest a reduction in muscle quality in individuals with type 2 diabetes mellitus and strengthen the reasons of investigating muscle quality rather then raw measures of muscle mass or muscle strength. Based on these data, change in muscle quality was used in the current study and calculated as follows: [(peak torque post of right leg / lean mass post of right leg)–(peak torque pre right leg / lean mass pre of right leg)][5].

#### Statistical analysis

Baseline data are presented as mean ± standard deviation and categorical variables are presented as N and (%). A one-way ANOVA was performed to identify baseline differences among groups of intervention as well as between tertiles of changes in muscle quality for continuous variables. A Chi square test was used to assess baseline differences for categorical variables among tertiles of changes in muscle quality.

Pearson’s correlations were used to quantify the association between changes in muscle quality and change in CRF measures. Multiple linear regression models were used to investigate the independent association between continuous changes in muscle quality and change in CRF measures following 9 months of exercise training. Multiple linear regression models were used to determine trends (P-trend) across tertiles of changes in muscle quality. Control group was coded as a single group and compared to intervention group to investigate the impact of change in muscle quality on CRF measures. Change (post-pre values) in CRF measures following exercise training are presented as adjusted least squared means (95% CIs). All analyses were adjusted for age, sex, ethnicity, type 2 diabetes mellitus duration, groups of intervention, and baseline value. Statistical significance was determined at P≤ 0.05 (2-tailed). All analyses were performed using SAS version 9.3 (SAS Institute Inc, Cary, North Carolina).

## Results

### Participant Characteristics

Our sample of individuals with type 2 diabetes mellitus (HbA_1c_: 7.2 ± 1.1%; 55.0 ±12.0 mmol/mol) had a mean age of 57.5 ± 8.0, BMI of 34.3 ± 5.8 kg/m^2^, and a VO_2peak_ of 19.4 ± 4.3 ml·kg^-1^·min^-1^·. Most of our participants were women (63.7%), non-Hispanic white (56.6%) and non-smokers (64.7%).

### Participant Characteristics across Interventions and Tertiles of Change in Muscle Quality

At baseline, with the exception of ethnicity (p = 0.044), no significant differences were observed among groups for age, sex, total and right leg lean body mass, muscle strength, and muscle quality (all p >0.05; Table 1). Nevertheless, there was a significant difference among groups of interventions for change in muscle quality (p = 0.0003).

**Table 1:** General characteristics.

	Control (N = 34)	AT (N = 49)	RT (N = 56)	ATRT (N = 57)	P values
**General Characteristics**					
Age (years)	59.1 ± 8.3	56.0 ± 7.8	58.3 ± 8.3	57.0 ± 7.8	0.270
Male (%)	11 (32.3)	17 (34.6)	23 (41.0)	20 (35.0)	0.832
**Race/Ethnicity (%)**					
Non-Hispanic White	19 (55.8)	30 (61.2)	32 (57.1)	30 (52.6)	0.848
African-American	13 (38.2)	19 (38.7)	23 (41.0)	21 (36.8)	0.974
Hispanic/other	2 (5.8)	0 (0.0)	1 (1.7)	6 (10.5)	0.044
**Smoking (%)**					
Current	1 (2.9)	0 (0.0)	1 (1.7)	1 (1.7)	0.740
Former	11 (32.3)	13 (26.5)	19 (33.9)	15 (26.3)	0.766
**Body composition**					
Weight (kg)	95.9 ± 21.1	94.3 ± 14.6	96.7 ± 15.6	97.7 ± 19.0	0.792
BMI (kg/m^2^)	34.4 ± 6.3	33.7 + 5.5	34.2 ± 5.4	34.9 ± 6.1	0.745
Waist circumference (cm)	109.2 ± 14.4	108.8 ± 11.8	111.4 ± 12.0	112.4 ± 13.4	0.436
Body fat (%)	38.4 ± 6.9	37.1 ± 7.7	37.4 ± 7.7	38.5 ± 7.0	0.711
Total fat mass (kg)	37.5 ± 12.0	35.5 ± 9.5	36.7 ± 10.3	38.4 ± 11.6	0.536
Trunk fat mass (kg)	19.3 ± 6.8	18.8 ± 4.8	19.8 ± 5.2	20.6 ± 6.1	0.412
Total lean body mass (kg)	56.3 ± 12.0	57.0 ± 10.8	58.0 ± 10.4	57.5 ± 11.5	0.914
Total right leg lean body mass (kg)	9.9 ± 2.2	9.7 ± 1.9	9.8 ± 1.8	9.8 ± 2.1	0.966
**Exercise test variables**					
Absolute VO_2 peak_ (L/min)	1.7 ± 0.5	1.9 ± 0.5	1.9 ± 0.5	1.8 ± 0.4	0.450
Relative VO_2 peak_ (mL·kg^-1^·min^-1^)	18.4 ± 3.8	20.5 ± 5.2	19.6 ± 4.4	18.8 ± 3.4	0.126
Treadmill time to exhaustion (min.)	10.8 ± 2.1	11.1 ± 3.0	10.5 ± 2.5	10.9 ± 2.3	0.686
Estimated METs	6.6 ± 1.3	7.2 ± 1.6	6.9 ± 1.3	6.7 ± 1.2	0.242
Isokinetic leg muscle strength (Nm)	123.4 ± 40.3	132.6 ± 46.1	131.3 ± 46.3	127.6 ± 48.1	0.801
Muscle quality (Nm^-1.^kg^-1^)	12.4 ± 2.8	13.6 ± 3.9	13.2 ± 3.6	12.8 ± 3.5	0.434
‡Change in muscle quality (Nm^-1.^kg^-1^)	0.39 ± 2.2	-0.49 ± 2.3	1.3 ± 2.1	0.95 ± 2.2	0.0003
**Diabetes**					
HbA_1c_ (%)	7.5 ± 1.4	7.0 ± 0.9	7.0 ± 0.9	7.2 ± 1.1	0.118
Diabetes duration (years)	6.8 ± 4.9	7.5 ± 5.9	7.5 ± 5.8	7.0 ± 5.6	0.916

Baseline continuous and categorical variables are presented as mean ± standard deviation and N (%);

^‡^ Continuous change in muscle quality (Post-Pre);

AT = Aerobic Training, RT = Resistance Training, ATRT = combination of Aerobic Training and Resistance Training, BMI = Body mass index, LBM = Lean body mass, METs = Metabolic equivalents tasks, HbA_1c_ = Hemoglobin A_1c_

Similarly, no differences were observed at baseline for age, sex, ethnicity, adiposity measures, total and right leg lean body mass, muscle strength, muscle quality, CRF, and HbA_1c_ across tertiles of change in muscle quality (all p > 0.05; Table 2). However, there was a significant difference for change in muscle quality (p< 0.0001) and the proportion of individuals in each group of intervention (p = 0.006) across tertiles of change in muscle quality.

**Table 2:** General Characteristics Stratified by Tertiles of Change in Muscle Quality.

		Tertiles of change in Muscle Quality			P-Values
	Overall (N = 196)	Tertile1 (n = 65) -1.82 (-2.1; -1.5)	Tertile2 (n = 66) 0.49 (0.4; 0.6)	Tertile3 (n = 65) 3.2 (2.8; 3.5)	
**General Characteristics**					
Age (years)	57.5 ± 8.0	57.2 ± 8.1	58.5 ± 8.1	56.9 ± 8.0	0.472
Men (%)	71 (36.2)	22 (33.5)	25 (37.9)	24 (36.9)	0.882
Group C/AT/RT/ATRT (n)	34/49/56/57	14/24/13/14	12/16/15/23	8/9/28/20	0.006
**Race/Ethnicity (%)**					
White Non-Hispanic	111 (56.6)	36 (55.4)	39 (59.1)	36 (55.4)	0.884
African American	76 (38.7)	27 (41.5)	23 (34.8)	26 (40.0)	0.712
Hispanic/Other	9 (4.5)	2 (3.1)	4 (6.1)	3 (4.6)	0.716
**Smoking (%)**					
Current	3 (1.5)	1 (1.5)	1 (1.5)	1 (1.5)	0.998
Former	58 (29.5)	18 (27.7)	18 (27.7)	22 (33.8)	0.654
**Body composition**					
Weight (kg)	96.3 ± 17.4	98.2 ± 17.0	95.8 ± 17.3	95.0 ± 18.0	0.516
BMI (kg/m^2^)	34.30 ± 5.8	35.0 ± 6.2	34.0 ± 5.4	34.0 ± 5.8	0.549
Waist circumference (cm)	110.7 ± 12.8	111.5 ± 12.5	110.5 ± 13.3	110.1 ± 13.0	0.829
Body fat (%)	37.8 ± 7.3	38.2 ± 7.5	37.7 ± 7.9	37.7 ± 6.7	0.926
Fat mass (kg)	37.0 ± 10.8	38.2 ± 11.4	36.7 ± 11.1	36.1 ± 9.8	0.550
Trunk fat mass (kg)	19.7 ± 5.7	20.1 ± 5.8	19.6 ± 6.1	19.6 ± 5.2	0.863
Lean body mass (kg)	57.3 ± 11.0	58.0 ± 10.1	57.1 ± 10.8	57.1 ± 10.8	0.844
Total right leg lean body mass (kg)	9.8 ± 1.9	9.9 ± 1.7	9.8 ± 1.9	9.8 ± 2.2	0.938
**Exercise test variables**					
Absolute peak VO_2_ (L^-1.^ min^-1.^)	1.8 ± 0.5	1.9 ± 0.5	1.8 ± 0.5	1.8 ± 0.5	0.708
Relative peak VO_2_ (ml·kg^-1^·min^-1^·)	19.4 ± 4.3	19.6 ± 4.5	19.5 ± 4.6	19.3 ± 4.0	0.909
Treadmill time to exhaustion (min.)	10.8 ± 2.5	10.9 ± 2.5	10.8 ± 2.8	10.8 ± 2.4	0.973
Estimated METs	6.9 ± 1.3	6.8 ± 1.5	6.9 ± 1.4	6.9 ±1.3	0.926
Isokinetic leg muscle strength (Nm)	129.2 ± 45.7	134.2 ± 44.9	131.1 ± 37.6	122.3 ± 53.5	0.308
Muscle quality (Nm^-1.^kg^-1^)	13.1 ± 3.5	13.5 ± 3.7	13.4 ± 3.0	12.4 ± 3.8	0.117
**Diabetes**					
HbA_1c_ (%)	7.2 ± 1.1	7.0 ± 1.0	7.2 ± 1.0	7.3 ± 1.3	0.407
Diabetes duration (years)	7.2 ± 5.6	7.3 ± 5.9	7.4 ± 5.5	7.2 ± 5.5	0.975

Baseline continuous and categorical variables are presented as mean ± SD and N (%).

### Bivariate Associations between Change in Muscle Quality and CRF Measures

Change in right leg lean body mass was not associated with any of the CRF measures (r range between -0.10 to 0.10; p = 0.200 to 0.390). Change in muscle strength was only associated with change in absolute (r = 0.18; p = 0.010), and relative (r = 0.16; p = 0.024) VO_2peak_, but not with TTE (r = 0.11; p = 0.116) or estimated METs (r = 0.06; p = 0.392).

Change in muscle quality after 9 months of exercise training was positively associated with change in absolute (r = 0.15; p = 0.034), and relative (r = 0.18; p = 0.012) VO_2peak_, as well as TTE (r = 0.14; p = 0.056). However, no such associations were observed with change in estimated METs (r = 0.09; p = 0.179;Table 3).

**Table 3:** Correlations between Changes in Muscle Quality and Exposure Variables following 9-month of Exercise Training.

	r	P Values
**Fitness Measures**		
Absolute peak VO_2_ (L^-1.^ min^-1.^)	0.15	0.034
Relative peak VO_2_ (ml·kg^-1^·min^-1^·)	0.18	0.012
Treadmill time to exhaustion (min.)	0.14	0.056
Estimated METs	0.09	0.179

Pearson correlations are presented as r and P value.

### Independent Associations between Change in Muscle Quality and CRF Measures

Continuous change in muscle quality was positively associated with change in absolute (β = 0.015; p = 0.019; R^2^ = 11.1%), and relative (β = 0.200; p = 0.005; R^2^ = 10.8%) VO_2peak_, as well as TTE (β = 0.170; p = 0.043; R^2^ = 8.4%) following 9 months of exercise training independent of age, sex, ethnicity, type 2 diabetes duration, and baseline value. However, no association was found between change in muscle quality and change in estimated METs (β = 0.044; p = 0.154; R^2^ = 9.8%) following 9 months of exercise training in individual with type 2 diabetes mellitus (Table 4). Further adjustment for group of intervention showed that change in muscle quality was significantly associated with improvement in absolute (β = 0.012; p = 0.050), and relative (β = 0.171; p = 0.016) VO_2peak_. No association was observed for change in muscle quality with TTE (β = 0.114; p = 0.168) and estimated METs (β = 0.028; p = 0.362).

**Table 4:** Linear Association between Changes in Muscle Quality and Changes in Exposure Variables following 9-Month of Exercise Training.

	Β	SE	P Values
**Fitness Measures**			
Absolute peak VO_2_ (L^-1.^ min^-1.^)	0.015	0.01	0.019
Relative peak VO_2_ (ml·kg^-1^·min^-1^·)	0.198	0.07	0.005
Treadmill time to exhaustion (min.)	0.169	0.08	0.043
Estimated METs	0.044	0.03	0.154

Data are presented as Beta and SE.

*The multiple linear regressions* are adjusted for age, sex, ethnicity, diabetes duration, and baseline values.

When analyses were restricted to the exercisers only, change in muscle quality was independently associated with change in absolute (β = 0.014; p = 0.050) and relative (β = 0.180; p = 0.019) VO_2peak_. No such independent association was observed for change in muscle quality and change in TTE (β = 0.162; p = 0.079) or estimated METs (β = 0.042; p = 0.218). Moreover, the models were further adjusted for group of intervention; change in muscle quality was independently associated with change in relative (β = 0.160; p = 0.045) VO_2peak_.

### P-Trend Analyses across Tertiles of Change in Muscle Quality and CRF Measures

**Fig 2:**
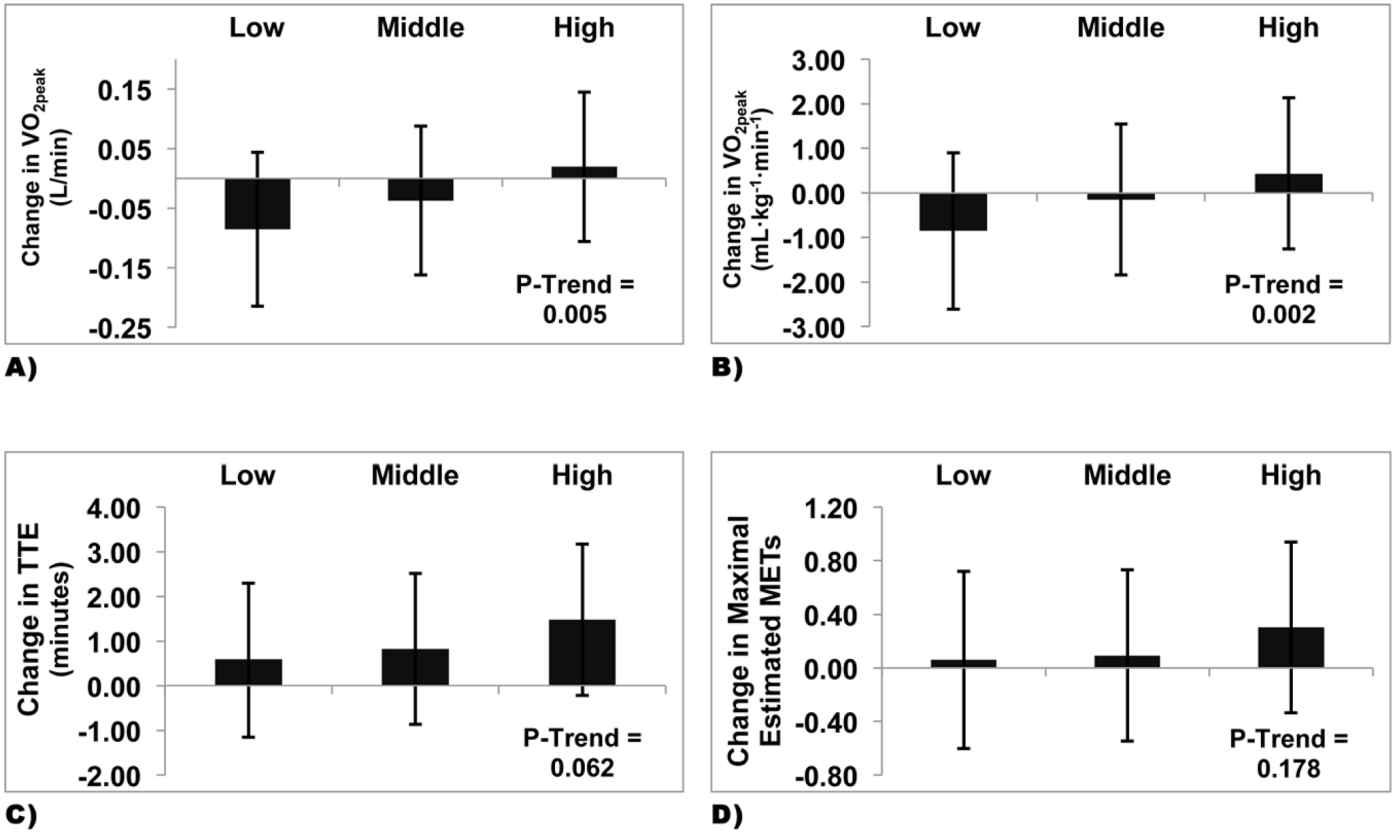
Change in Fitness Level Across Tertiles of Changes in Muscle Quality in Individuals with Type 2 Diabetes Mellitus. 2A. P-trend analyses of change in absolute VO_2peak_ across tertiles of change in muscle quality. 2B. P-trend analyses of change in relative VO_2peak_ across tertiles of change in muscle quality. 2C. P-trend analyses of change in treadmill time to exhaustion across tertiles of change in muscle quality. 2D. P-trend analyses of change in maximal estimated METs across tertiles of change in muscle quality. Fig 2A–2D. Data are are presented as lsmeans (95%) confidence intervals.

A significant trend was observed across tertiles of change in muscle quality (Fig 2A and 2B) for changes in absolute (β = 0.050; p = 0.005) and relative (β = 0.624; p = 0.002) VO_2peak_ following 9 months of exercise training. Although it did not reach significance, a trend was observed across tertiles of change in muscle quality with the greatest increase in TTE in the highest tertiles of change in muscle quality (β = 0.451; p = 0.0623) (Fig 2C). However, no trends were observed for changes in estimated METs (β = 0.122; p = 0.178) across tertiles of change in muscle quality after 9 months exercise (Fig 2D). Further adjustment for group of intervention revealed a significant trend across tertiles of change in muscle quality and change in absolute (β = 0.043; p = 0.019) and relative (β = 0.539; p = 0.009) VO_2peak_. No such trend was observed across tertiles of change in muscle quality and change in TTE (β = 0.274; p = 0.253) and estimated METs (β = 0.070; p = 0.442).

Analyses were restricted to exercisers only and a significant trend was observed across tertiles of change in muscle quality for change in absolute (β = 0.043; p = 0.040) and relative (β = 0.510; p = 0.028) VO_2peak_. However, no such trends were observed across tertiles of change in muscle quality and TTE (β = 0.358; p = 0.196) and estimated METs (β = 0.110; p = 0.287).

### Impact of Intervention Groups and Muscle Quality Groups on CRF Measures

**Fig 3:**
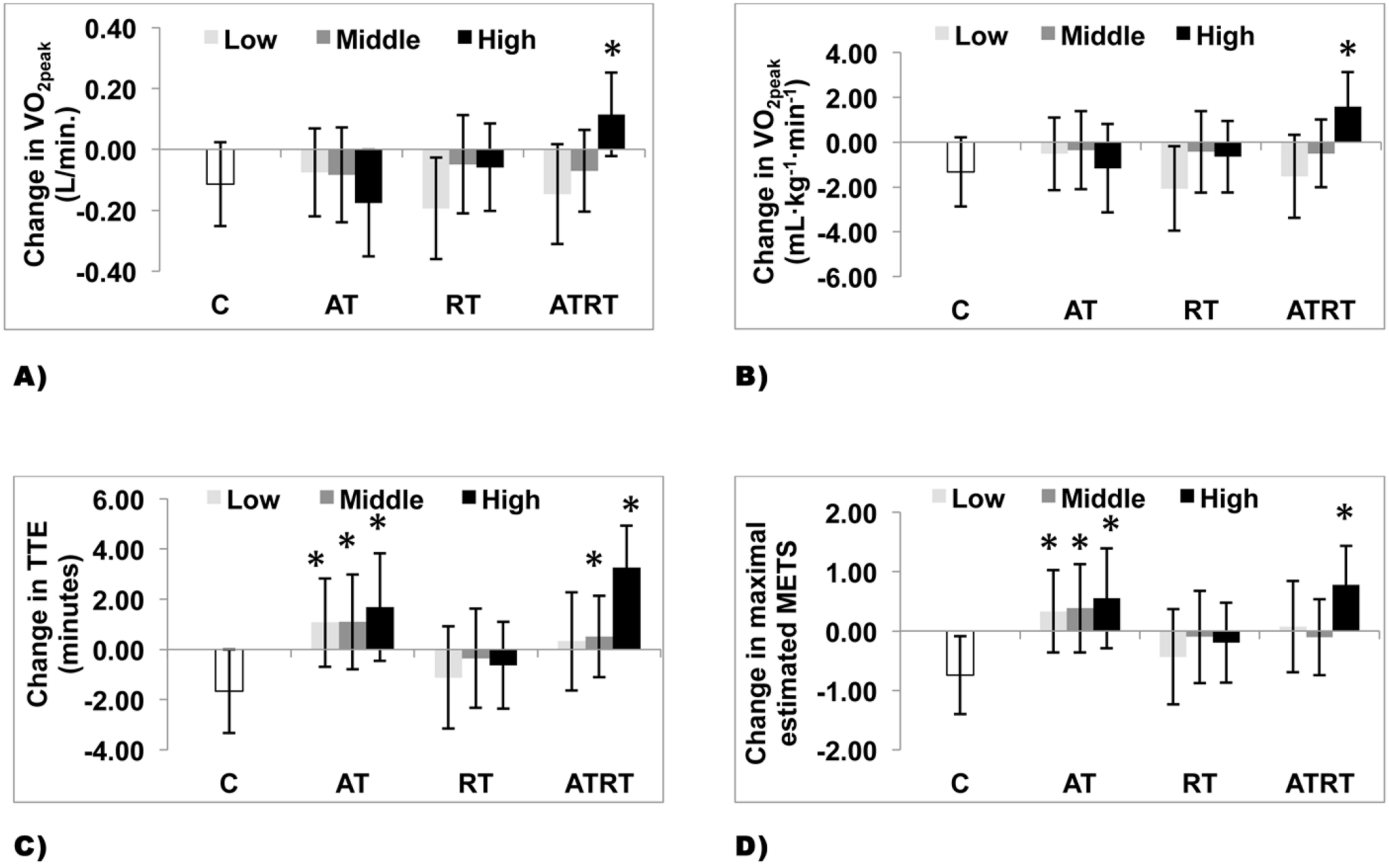
Change in Fitness Level by Group of Intervention and Tertiles of Changes in Muscle Quality in Individuals with Type 2 Diabetes Mellitus. 3A. Change in absolute VO_2peak_ across group of intervention and tertiles of change in muscle quality. 3B. Change in relative VO_2peak_ across group of intervention and tertiles of change in muscle quality. 3C. Change in treadmill time to exhaustion across group of intervention and tertiles of change in muscle quality. 3D. Change in maximal estimated METs across group of intervention and tertiles of change in muscle quality. Fig 3A–3D. Data are presented as lsmeans (95%) confidence intervals. * P< 0.05 significantly different from the control group.

Main effects were observed for change in muscle quality with change in absolute (p = 0.056), relative (p = 0.021) VO_2peak_, and TTE (p = 0.022), while no main effect was observed for change in estimated METs (p = 0.112) (Fig 3A–3D). Moreover, significant interaction between tertiles of change in muscle quality and group of interventions were observed for absolute (p = 0.006), relative (p = 0.013) VO_2peak_ and TTE (p = 0.049), while no interaction was observed for estimated METs (p = 0.227).

Further analyses were performed with the control group coded as a single group. A significantly greater improvement in absolute (p = 0.0007) and relative (p = 0.0001) VO_2peak_, TTE (p = 0.0001), and estimated METs (p = 0.0001) were observed in the highest tertile of change in muscle quality in the ATRT group compared to the control group. Individuals in the AT group improved TTE (p< 0.05) and estimated METs (p< 0.01) independent of change in muscle quality tertiles.

## Discussion

The primary result of this study is that change in muscle quality following 9 months of exercise training was positively and independently associated with an increase in CRF measured by absolute and relative VO_2peak_ in individuals with type 2 diabetes mellitus. In addition, we found that individuals with type 2 diabetes mellitus who performed both AT and RT and had the greatest increase in muscle quality significantly improved CRF measures (absolute and relative VO_2peak_, TTE, and estimated METs) compared to the control group. Collectively, these data shed light on factors that may contribute to improved CRF measures through exercise training in individuals with type 2 diabetes mellitus and which may help to design future intervention studies to manage risk factors, in particular low CRF, in individuals with type 2 diabetes mellitus.

In the present study, we observed a linear trend for improvement in CRF and TTE in individuals with type 2 diabetes mellitus across tertiles of change in muscle quality. These results have clinical implications, since a prospective study showed that individuals with moderate or high CRF had 38% and 63% lower risk, respectively, of developing type 2 diabetes during 18 years follow-up[27]. Similarly, prospective data showed a steep inverse association between CRF and premature mortality in adults with[17, 18] or without type 2 diabetes mellitus [24, 28]. Additionally, evidence suggests that maintaining a higher CRF in individuals with type 2 diabetes mellitus has been associated with a decrease in CVD risk factors and the risk of premature CVD mortality[16, 24, 28–30]. This association appears to be independent of adiposity as a study showed that individuals with type 2 diabetes mellitus with low CRF was associated with higher risk of CVD mortality in normal weight (2.7-fold), overweight (2.7-fold), and obese individuals (2.8-fold)[24]. Moreover, studies suggest a 10–15% reduction in CVD-coronary diseases mortality and a 7.9–13% reduction in all-cause mortality for each increase of 1-ml·kg^-1^·min^-1^· or 1-METs[12, 31, 32]. Based on our results, 41.8% of our sample increased their CRF level by at least 1-ml·kg^-1^·min^-1^· and/or 1-METs following exercise training. This increase in CRF level might have a significant impact on CVD-coronary and all-cause mortality risk reduction. Interestingly, individuals with type 2 diabetes mellitus who experience the greatest increase in muscle quality following an exercise intervention would have a 12.0% reduction in all-cause mortality, respectively.

In the HART-D study, the greatest increase in relative VO_2peak_ was observed in the ATRT group[19]. Results of the current study are in line with previous observations and add to the literature that individuals with type 2 diabetes mellitus performing ATRT and increase muscle quality are more likely to improve their CRF. This result is of considerable interest for the management of type 2 diabetes mellitus, as it suggests adding a RT component to an exercise intervention in order to enhance CRF of individuals with type 2 diabetes mellitus. In fact, the proportion of individuals that increased VO_2peak_ by at least 1ml·kg^-1^·min^-1^ was 26.3%, 18.8%, and 6.1% in the ATRT, RT, and AT group who had the greatest change in muscle quality. This is clinically relevant considering an increase as low as 0.7ml·kg^-1^·min^-1^· was associated with a 30% reduced mortality risk[32]. Additionally, these improvements in CRF with RT are noteworthy, as muscle strength itself is known to be associated with favorable CVD risk factors and survival[33, 34]. The impact of muscle quality on CRF is unclear; however our group showed in a sub-sample of the HART-D, that ATRT improved several muscle enzymes, substrate oxidation and enhanced mitochondria biogenesis in individuals with type 2 diabetes mellitus, which could potentially explain our results[35]. In addition, a study showed that RT alone significantly improved mitochondria biogenesis in older adults[36], while another study compared the impact of RT alone in young and older adults on mitochondria biogenesis and found a significant improvement in older adults only[37]. Therefore, it is possible that our participants, with a mean age of 57.5 ± 8.0 years and with type 2 diabetes mellitus, in the HART-D study benefits from a synergetic effect of ATRT on mitochondria potential. To our knowledge, this study is the first randomized controlled trial suggesting that improvement in muscle quality, as determined by a ratio of strength to muscle mass, is associated with improvement in CRF in individuals with type 2 diabetes mellitus.

The main limitation of the present study is the lack of gold standard measures of muscle quality as determined by computed tomography or magnetic resonance imaging. Our study, however, is strengthened by the tightly controlled exercise intervention, which allows us to have a high percentage of exercise compliance (70%). Additionally, muscle quality has been measured with DXA scan and Biodex, two gold standard technologies to assess body composition and muscle strength. Finally, our sample was heterogeneous in terms of age, sex, and ethnicity, which strengthened our study by increasing external validity, and the randomized controlled trial design strengthens our observation. As of now, only one exercise trial has investigated the impact of change in muscle quality in individuals with type 2 diabetes[9]. In this study, sixty-two Hispanic individuals were randomized to a 16-week of RT or a control group. Following the intervention, significantly greater improvement in muscle quality and whole-body insulin sensitivity was observed in the RT group compared to a control group. However, this study did not quantify the impact of change in muscle quality on CRF, which is novel in our study. Finally, this muscle quality quantification is relevant and allows for surrogate measure of changes in muscle quality without using more sophisticated measures that are costly, have larger amount of radiation, and are more invasive.

In conclusion, exercise training induced increases in muscle quality was associated with a greater CRF in individuals with type 2 diabetes mellitus. In addition, an exercise intervention composed of AT and RT should be recommended to individuals with type 2 diabetes mellitus to increase muscle quality and CRF. Future research should further investigate the influence of changes in muscle quality in individuals with type 2 diabetes mellitus on other CVD risk factors, and explore the impact of a greater RT stimulus in this population and its impact on muscle quality and performance.

## Supporting Information

S1 CONSORT Checklist This is the CONSORT Checklist. (DOC)

S1 Protocol This is the protocol of the study. (PDF)
