# Supplementary material for: Association between Changes in Muscle Quality with Exercise Training and Changes in Cardiorespiratory Fitness Measures in Individuals with Type 2 Diabetes Mellitus: Results from the HART-D Study
Source: PLoS One. 2015 Aug 7;10(8):e0135057. doi: 10.1371/journal.pone.0135057 (PMC4529285; doi:10.1371/journal.pone.0135057)
Supplement: S1 Protocol — (PDF) [file pone.0135057.s002.pdf]

PBRC Institutional Review Board
FWA 00006218
Approved On 6/22/09
Signature [illegible]

Health Benefits of Aerobic and Resistance Training in individuals with type 2 diabetes (HART-D)

Timothy S. Church, MD, PhD, MPH, Principle Investigator
Conrad P. Earnest, PhD, Co-Investigator
William T. Cefalu, MD, Co-Investigator

Project Summary

The goal of the proposed study, Health Benefits of Aerobic and Resistance Training in individuals with type 2 diabetes (HART-D), is to compare the effect of resistance training alone (RT), resistance in combination with aerobic training (AT+RT), and aerobic training alone (AT) to stretching and relaxation (SR) on hemoglobin A1C (HbA1C), in initially sedentary women and men with type 2 diabetes (T2D). Although it is generally accepted that regular exercise provides substantial health benefits to individuals with T2D, the exact exercise prescription in terms of type (AT versus RT versus AT+RT) still remains largely unexplored, particularly in regard to week-to-week glucose control as assessed by HbA1C.

There is a need for more adequately powered and well-controlled studies to examine the effects of RT, AT and AT+RT on HbA1C in individuals with T2D. With the incidence of T2D expected to increase dramatically in the coming years, it is essential to have a better understanding of the relative benefits of various exercise interventions. This information can help better formulate exercise recommendations for patients with T2D as well as potentially provide more exercise options, which is important given the small percentage of individuals with TD2 who regularly exercise.

The study group will be sedentary women and men with T2D, aged 30 to 75 years. We will randomly assign 300 individuals to an aerobic exercise training only group (AT; n=87), a resistance training only group (RT; n=87), a combination of aerobic plus resistance training (AT+RT; n=87), or a stretching and relaxation group (SR; n=40). The SR individuals will complete one or more 45 minute sessions with a trained exercise professional that focus on increasing flexibility and reducing stress. This group is intended to serve as a control group, as we do not expect stretching to have measurable effects on HbA1C. The AT individuals will participate in 3 or 4 training sessions each week for 9 months progressing to a total energy expenditure of 12 kcal/kg/week (KKW), which is an exercise dose consistent with the current public health recommendations for physical activity for individuals with T2D.[1,2] The target exercise intensity will be 50%-80% of baseline VO_2 max. The RT group will participate in 3 sessions per week (9 exercises, 2-3 sets each), which focuses on large muscle groups. This RT regimen is based on the studies that most successfully improved HbA1C in individuals with T2D. Individuals in the AT+RT group will complete 10 KKW of aerobic training and a reduced resistance-training regimen of 2 sessions per week (9 exercises, 1 set of each). The AT+RT regimen represents the exercise recommendations of the American College of Sports Medicine (ACSM) and the American Diabetes Association (ADA).[3,4] All participants will complete a one hour consultation with a Certified Diabetes Educator (CDE) following randomization, during which, participants will be provided with educational materials and general guidelines for healthy living. Participants will also complete monthly sessions with the CDE, during which they will receive further instruction and guidance.

Simply stated, we wish to compare the effect of resistance training alone, resistance in combination with aerobic training, and aerobic training alone to stretching exercise on HbA1C, in initially sedentary women and men with T2D. The primary outcome measure is HbA1C, an integrated measure of blood glucose control over the past 8-12 weeks. Other outcomes of interest include homeostasis model assessment (HOMA), resting blood pressure, C-reactive protein (CRP), total body fat, and lean muscle mass as measured by DEXA, cardiorespiratory

fitness, muscular strength, quality of life, medication changes, and metabolic measures including serum cholesterol and triglycerides.

Background and Significance

T2D and Exercise (aerobic or resistance)-Morbidity and Mortality. Approximately 17 million individuals in the U.S. suffer from diagnosed or undiagnosed diabetes.[5] Of those with the disease, 90-95% have T2D. The estimated direct and indirect costs of the disease are $132 billion per year.[6] Individuals with T2D have at least twice the risk for premature death, heart disease, and stroke compared to individuals without T2D.[6] 73% of those with T2D also have high blood pressure.[6]

Many of the complications associated with T2D can be prevented through the adoption and maintenance of lifestyle behaviors such as regular exercise. The benefits of regular exercise in those with T2D are similar to the benefits in healthy individuals and improve T2D-specific variables.[7] The positive effects of aerobic and resistance training include improvements in glycemic control, insulin sensitivity, and cardiovascular risk factors.[8-10] Despite this, 31% of all individuals with T2D report no regular physical activity and another 38% report levels lower than those recommended for eliciting health benefits.[11] Lack of participation in physical activity by these individuals may be in part due to the lack of activity options combined with limited clinical trial derived exercise recommendations beyond those for aerobic exercise alone.[12]

Specific Aims

Specific Aim 1. **We will identify, recruit, assess, and randomly assign 300 sedentary individuals with diagnosed T2D to an aerobic training group, a resistance training group, a combination of aerobic plus resistance training group, or a standard care group to test the hypotheses that:**

- After 9 months, individuals randomized to the AT+RT group will have a greater average improvement in HbA1C than (a) individuals in AT group, and (b) individuals in RT group, and all exercisers will have great improvement in HbA1C than individuals in the SR group.
- After 9 months, individuals randomized in the RT group will have comparable changes in HbA1C to those individuals in the AT group.

Additional Specific Aims

In addition to the primary aims, we will evaluate the effects of SR, AT, RT, and AT+RT on several important secondary outcomes, including HOMA, resting blood pressure, CRP, total body fat and muscle mass, cardiorespiratory fitness and muscular strength, quality of life, medication changes, and metabolic measures including serum cholesterol and triglyceride.

HbA1C and Exercise

HbA1C: In review, hemoglobin A is the major form of hemoglobin in adults. HbA1C is a sub-species formed by the covalent bond between glucose and hemoglobin A. The formation of HbA1C is completely dependent on the concentration of blood glucose. In contrast to fasting glucose, which reflects blood glucose concentration at the time of blood draw, HbA1C is not acutely influenced by very recent changes in behavior, such as engaging in physical activity or taking recent medication. HbA1C concentration is a reflection of the average glucose concentration over an 8- to 12-week period and is used clinically to assess long-term glucose control. HbA1C concentration is strongly associated with future risk of morbidity and mortality in individuals with T2D, and improvements in HbA1C are associated with reduced risk for mortality.[13, 14] HbA1C is a cornerstone in clinical management of T2D, and quantifies long-term glucose control as opposed to recent (1-3 days) changes in medication or physical activity. Therefore, we have selected HbA1C as our primary outcome.

Figure 1. Mechanisms by which exercise training may improve insulin action and the control of blood glucose in T2D .

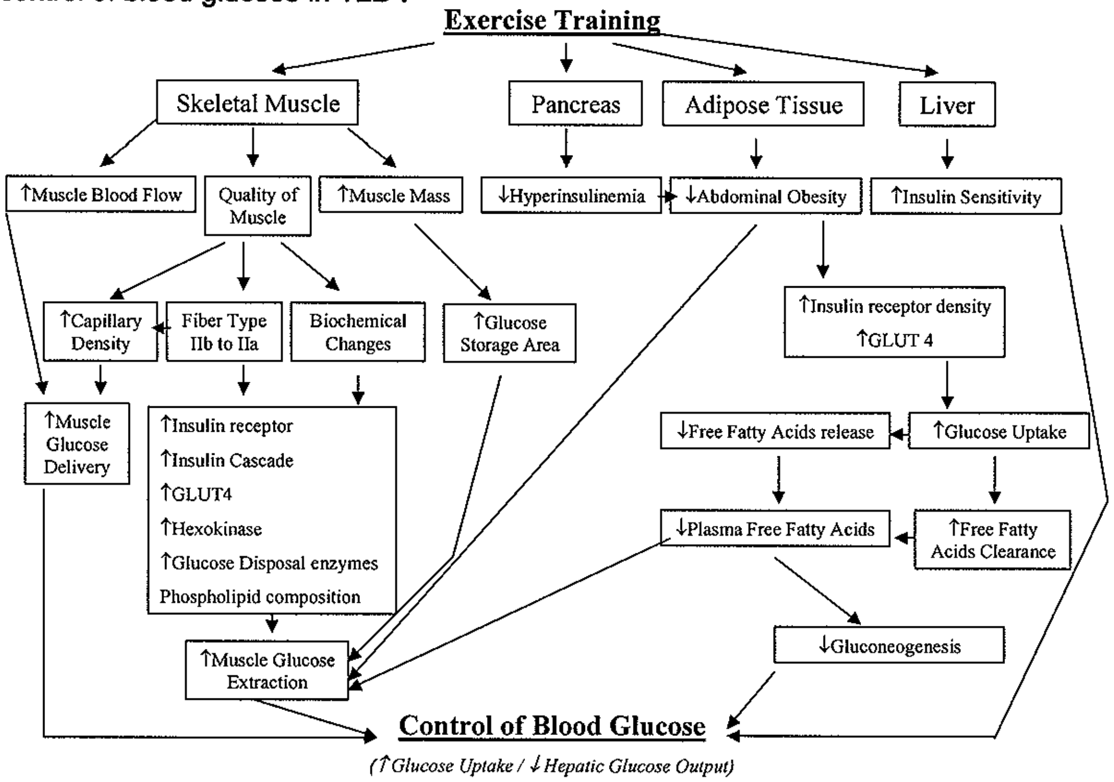

HbA1C and Aerobic Training: Although there is strong evidence that aerobic exercise provides substantial health benefits in individuals with T2D, the data to support improvements in HbA1C following aerobic exercise training are surprisingly weak. There is conflicting evidence on the effect of aerobic training on HbA1C. In a meta-analysis by Boule et al.[15] consisting of 12 aerobic training studies and 2 resistance training studies, exercise reduced HbA1C by 0.66%. However, many of the included studies had significant weaknesses such as a lack of randomized groups, small sample size (exercise groups n<20), and short study duration. Because HbA1C is a measure of average blood glucose concentration over an 8- to 12-week period, studies where the duration of training was 13 weeks or less (8 of 11 studies) may underestimate the effect of aerobic training on the concentration of HbA1C. Only one study had a trial period longer than 18 weeks. In summary, though the data are limited to a number of small studies, it is widely accepted that aerobic training may improve HbA1C concentration in individuals with T2D.

HbA1C and Resistance Training: The available studies pertaining to resistance training and HbA1C conflict and are deficient in number and quality. Two studies report no improvement in HbA1C with resistance training.[16, 17] However, these studies had small samples sizes (9 exercisers), and were of short duration (5-10 weeks). Two longer studies (24 and 16 weeks, respectively) reported decreases of 1.2% and 1.1% in HbA1C, respectively.[18, 19] Unfortunately, due to methodological insufficiencies these two studies provide little help in quantifying the independent effect of resistance training on HbA1C. For example, Dunstan et al [20] included weight loss in the intervention, and in a study by Castaneda et al [21] the resistance training group greatly increased their daily physical activity outside the study, which consisted almost entirely of aerobic activity, while the control group did not. Taken as a whole, these studies suggest resistance training may improve HbA1C in individuals with T2D. However, there is a need for an adequately powered, well-controlled study of long duration examining the effect of resistance training alone on HbA1C in individuals with T2D.

HbA1C and Combination Training: To our knowledge, no reports compare the changes in HbA1C concentration in combined aerobic and resistance training to either of these training

modes alone. The Castaneda et al [22] study, however, is provocative. Though designed to be a resistance training only study, the resistance-training group increased their leisure time physical activity from 23 min per week to 120 min per week (>500%), while the control group decreased their activity (-44%, p=0.001 for between group differences). The decrease in HbAIC in the training group, -1.2%, is difficult to interpret due to the confounding effect of increased physical activity. However, this decrease supports the hypothesis that combined aerobic and resistance training may be more beneficial than either one alone. It is noteworthy that despite a lack of trials examining combined aerobic with resistance training, this remains the exercise recommendation from ADA and ACSM.[23, 24]

Homeostasis Model Assessment of Insulin Resistance (HOMA): HOMA index has been used as an index of insulin resistance in T2D patients in the previous reports.[25, 26] It has been shown that HOMA is a reliable index of insulin sensitivity during the clinical course of patients with T2D. [27] HOMA is independently associated with risk of CVD outcomes.[28] The efficacy studies of exercise training to reduce HOMA are limited. One study showed a decrease in HOMA after 2 months of endurance exercise intervention in individuals with prediabetes.[29] On the contrary Helge reported decreases in HOMA index only when exercise training was combined with a carbohydrate-rich diet.[30] Miyatake et al[31] reported that increases in cardiorespiratory fitness after exercise intervention are accompanied by improvement in HOMA index. Additionally, no studies report the effect of aerobic and resistance training on HOMA levels in individuals with T2D. Thus, there is need for additional well-controlled exercise studies examining the effect of regular exercise on homeostasis model assessment of insulin resistance in individuals with T2D.

Blood Pressure: Essential hypertension is independently associated with T2D and its prevalence is estimated to be up to 73% in individuals with T2D.[32] For these individuals, concurrent hypertension markedly increases the risk of CVD morbidity and mortality.[33] The efficacy of aerobic training to lower blood pressure in diabetic individuals is not as well characterized or understood as it is in non-diabetic populations.[34] Even less is known about the benefits of resistance training (with and without aerobic training) on blood pressure in these individuals. Blood pressure control is critical for preventing morbidity and mortality in diabetic populations, and having a better understanding of the effect of aerobic, resistance, and combination training on blood pressure regulation in individuals with T2D is of great public health importance.

Lipids: Reviews on the relation of physical activity to blood lipid and lipoprotein levels include only a few studies with T2D individuals.[35] Cross-sectional observations show a direct dose-response relation between amount of habitual physical activity and high density lipoprotein cholesterol (HDL-C) levels.[36] There is consistent evidence that exercise training significantly reduces elevated levels of fasting and postprandial triglycerides in individuals with elevated fasting triglycerides, but has little or no effect on low density lipoprotein cholesterol (LDL-C) levels.[37-40] Exercise studies in individuals with T2D have discrepant results.[41-47] Possible reasons include an insufficient exercise dose and duration to evoke adaptation, study population differences, inadequate sample size, differences in the training regime, and variability in other lifestyle factors affecting blood lipids.

Inflammatory Markers: Elevated concentration of the inflammatory marker CRP is associated with increased risk of future development of T2D. CRP is inversely associated with glucose tolerance in individuals with T2D.[48] Although an elevated CRP concentration is an independent and strong risk factor for CVD events and mortality,[49-52] some investigators believe that data for a causal hypothesis are not compelling.[53] Even so, there are limited clinical therapies to improve elevated CRP. A number of cross sectional reports have found that physically active individuals have lower CRP concentrations compared with sedentary individuals.[54-57] Additionally, CRP may decrease after regular exercise training independent of weight loss.[58] We have published the largest study to date showing cardiorespiratory fitness, measured by maximal treadmill exercise test, to be inversely related to CRP in healthy men independent of body mass index.[59]

Summary and Importance of the Study

- An estimated 17 million Americans have diabetes with >90% being T2D, and this number is expected to grow.
- Epidemiological data, including our own work, supports the hypothesis that regular exercise greatly decreases the risks associated with T2D.
- HbA1C is a key summary measure of long-term glucose control in individuals with T2D and is associated with risk of future morbidity and mortality.
- Aerobic exercise is generally accepted to improve HbA1C.
- Resistance training is potentially beneficial for individuals with T2D. However, data examining the effect of resistance training on HbA1C has conflicts, and few studies use adequate control and randomization methods. Further, the strongest studies in support of resistance training modifying HbA1C are confounded by changes in weight and physical activity.
- The reductions in HbA1C attributed to aerobic and resistance training may be additive, or at least partly additive. This is supported by training specific physiological adaptations seen in participants who participated in resistance training and increased their physical activity. However, no study has adequately tested the additive effects in a prospective, randomized, controlled trial.

HART-D will be an adequately powered, randomized, controlled trial to test standard care, aerobic, resistance, or combination training on individuals with T2D. The focus is to compare AT+RT to AT and RT and RT to AT in lowering HbA1C concentration, a marker of long-term blood glucose control, while also assessing the efficacy of all forms of exercise relative to standard care. HART-D will make important contributions to our understanding of the effects of AT+RT and RT in individuals with T2D and help refine public health and clinical recommendations for this group. Individuals with T2D are at extremely high risk of adverse health outcomes and developing evidence-based exercise recommendations for them should be given a high priority on the national public health agenda.

Research Design and Methods

Overview. We will randomly assign 300 individuals to an SR, AT, RT, or an AT+RT group. The AT individuals will participate in 3 or 4 training sessions each week for 9 months at an energy expenditure of 12 KKW. The target exercise intensity will be 50%-80% of baseline VO_2 max. The SR group will complete one or more 45 minute sessions of light stretching exercises each week. The RT group will participate in 3 sessions per week (~45 minutes each), focusing on large muscle (9 exercises, 2-3 sets each). This RT regimen is based on previous small or uncontrolled studies that appear to have been successful in improving HbA1C in individuals with T2D.[65-67] Individuals in the AT+RT group will complete 10 KKW of aerobic training and a resistance-training regimen of 2 sessions per week (9 exercises, 1 set of each). This combination represents current exercise recommendations of the ADA and ACSM.[68, 69]

The primary outcome measure is HbA1C. Other outcomes of interest include HOMA index, resting blood pressure, CRP, total body fat and muscle mass, cardiorespiratory fitness, muscular strength, quality of life, medication changes, and metabolic measures including serum cholesterol and triglycerides. All the secondary outcomes have previously been found to be abnormal in individuals with T2D and are associated with increased risk of mortality and morbidity. The study will run for 4 years.

Study Participants. Study participants will be 300 women and men 30 to 75 years of age with T2D, with ≥ 25% of participants from minority groups. We selected inclusion/exclusion criteria to produce a study group of sedentary individuals with T2D who are willing and able to participate in 9 months of exercise training in a controlled trial. T2D is determined by self-report and verified through review of medical records, current treatment, verification from

personal health care provider, or documentation of fasting glucose ≥126 mg/dL. Individuals who have a clinical history strongly suggestive of Type 1 Diabetes will be excluded.

During screening and baseline assessment, individuals who have urgent medical conditions or values of HbA1C, triglycerides, cholesterol, creatinine, or blood pressure that exceed eligibility limits will be referred for medical care. Depending on the medical condition, those ineligible because of HbA1C, triglycerides, cholesterol, or blood pressure may be re-screened for eligibility.

Inclusion and Exclusion Criteria

Inclusion Criteria

- Age 30 to 75 years.
- Type 2 diabetes determined by self-report with verification (see text for details).
- 6.5% ≤ HbA1C ≥11%.
- Sedentary lifestyle: not being physically active ≥3 d/wk for 20 min each time for the previous 6 months, and not participating in regular resistance exercise.[70, 71]

Exclusion Criteria

- Inadequate control of co-morbid conditions.
- Resting blood pressure ≥160/100 mm Hg.
- Triglycerides ≥500 mg/dL.
- BMI > 48.
- Use of an insulin pump
- Factors that may limit adherence to intervention or affect conduct of the trial
  - Unable or unwilling to communicate with staff, to provide written informed consent, or accept the randomized assignment.
  - Failure to complete behavioral run-in and baseline testing.
  - Hospitalization for depression in the last 6 months.
  - Not physically capable of performing the exercise required of the study protocols.
  - Consuming >14 alcoholic beverages per week.
  - Plans to be away >4 weeks in the next 9 months.
  - Lack of support from primary health care provider or family members.
  - Significant weight loss in the past year (>20 lbs) or current use of weight loss medications.
  - Current diagnosis of schizophrenia, other psychotic disorders, or bipolar disorder.
  - Another member of household is a staff member of HART-D.
  - Bariatric surgery within last 3 years.
  - Other temporary intervening event, such as sick spouse, bereavement, or recent move.
  - Other medical, psychiatric, or behavioral limitations that, in the view of the principal investigator, may interfere with study participation or the ability to follow the intervention protocol.
- Underlying diseases or conditions likely to limit lifespan and/or affect the safety of the intervention
  - Pregnant or plan on becoming pregnant in the next 9 months.
  - Cancers requiring treatment in the past 5 years, unless prognosis is excellent.
  - Self-reported HIV, tuberculosis, Hepatitis B, or Hepatitis C.
  - History or evidence of serious arrythmias, cardiomyopathy, congestive heart failure, aortic aneurysm, or heart transplantation.
  - Renal disease: urine protein >100mg/dL, serum creatinine ≥ 1.5 mg/dL or currently receiving dialysis.
  - Auto-immune diseases (such as Lupus, Multiple Sclerosis, Graves' disease, or Rheumatoid arthritis).
  - Advanced neuropathy or retinopathy.
  - Chronic obstructive lung disease, peripheral vascular disease or angina that limits ability to follow exercise protocol.

- History of stroke
- History of vascular aneurysms
- Any other medical condition or disease that is life threatening or that can interfere with or be aggravated by exercise.

Study Flow.

Overall study flow is outlined below.

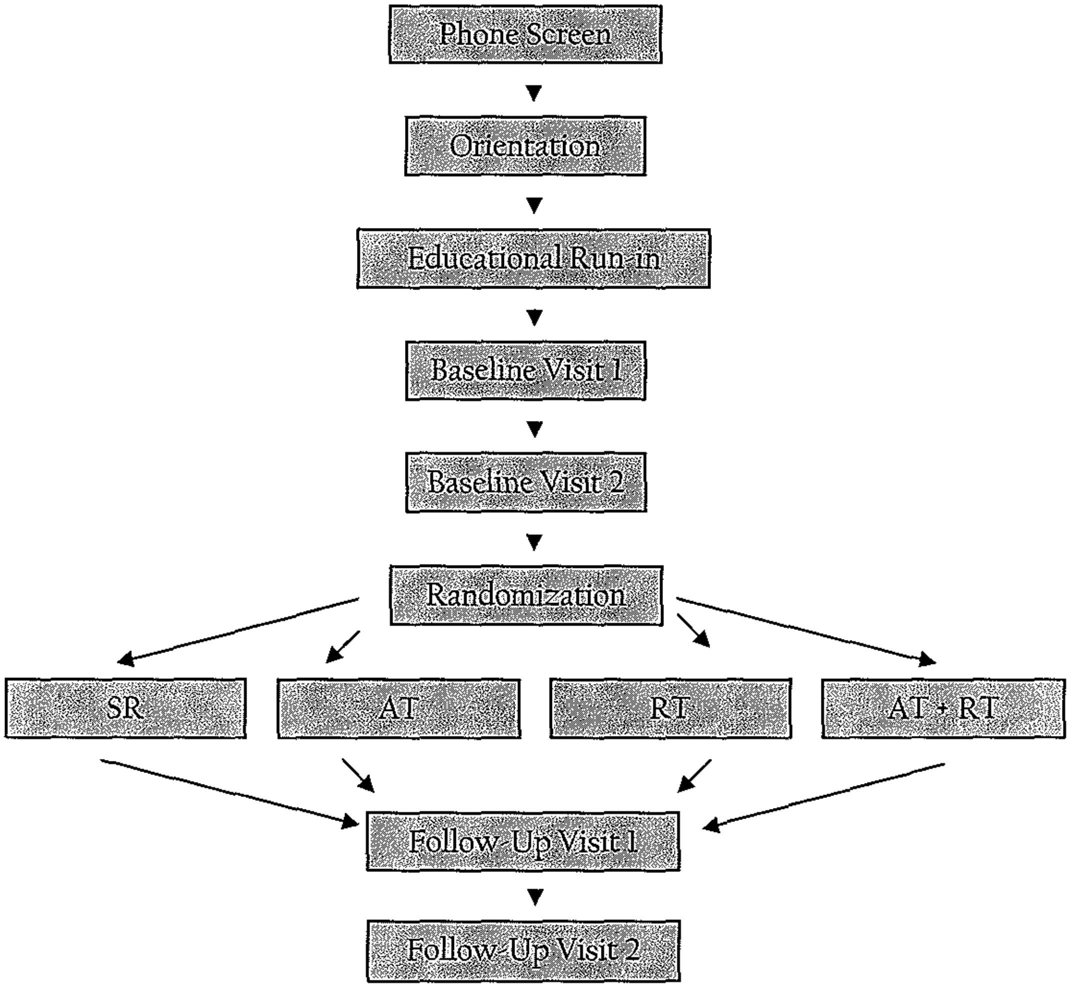

Recruitment.

Participants will be recruited via volunteer lists, the PBRC website, word-of-mouth and community outreach events. In addition, we will utilize local newspapers, radio and TV stations to recruit via the media. Other methods such as direct mailing, e-mail, low cost flyers placed around the community (shopping malls, supermarkets, etc), and advertising in corporate newsletters will also be used.

Telephone Prescreen. We will perform an initial telephone screen of individuals who respond to various recruitment strategies. We will ask current age, birth date, and questions regarding medical history. Questions include yes and no inquiries about physical conditions that might keep them from exercising, such as orthopedic limitations, listing current medications, and frequency of exercise. This survey can be quickly completed over the telephone. Eligible participants (i.e., sedentary individuals with T2D, between the ages of 30-75; who do not have any physical problem that might keep them from exercising) will be invited for an orientation session.

Orientation Session. During the orientation session the study will be explained in detail and participants will be given the chance to ask questions. Those who are still interested in participating in the study will be asked to sign an informed consent to continue.

Educational Run-In Sessions. All participants who are eligible after the orientation session will be invited to participate in a multi-visit run-in that will be a comprised of computer education programs. Since all individuals will be randomized to some form of exercise the run-in sessions will be focused on safety issues specific to individuals with T2D starting an exercise program. For example, the run-in session will address what to expect in terms of changes in blood glucose levels as a result of starting an exercise program, importance of checking for pressure sores or blisters, and tracking of medication regime including changes due to exercise. In addition to providing important safety education to the study participants, the run-in will test subjects' ability to come to PBRC 3 or more times a week, and we have found that this reduces early drop out in controlled trials. During this time, we will also perform basic anthropometric measures, a resting EKG, and medical exam to ensure that it is safe for participants to participate in HART-D and that they meet general inclusion criteria. After the participant successfully completes the educational run-in sessions, they will be scheduled for baseline evaluation.

Baseline and Follow-Up Assessments. All assessments will occur at PBRC. For the purposes of this application, assessment refers to the blood draws, exercise testing, and body composition. The baseline and 9-month follow-up assessments will take 2 visits. The composition and flow of the visits at baseline and follow-up will be nearly identical. The only exception being that at baseline after completion of all testing procedures participants will be randomized; while at follow-up participants will have an exit interview.

Randomization. Participants will be randomized after the project physician and project director have determined that the participant meets all criteria for enrollment and all baseline data are complete. Participants will meet one-on-one with a HART-D staff member in a private room. Each participant will then be enrolled via a web-based randomization program developed at Pennington Biomedical Research Center. The HART-D staff member will record each enrollment as it occurs. The project biostatistician will keep a secure record of the entire assignment sequence, which when combined with the participant tracking system will provide an audit trail.[72]

Rationale for Timing of Randomization. Randomization will take place following a run-in period and baseline testing, which will serve to exclude participants with poor adherence. We recognize that some clinical trials randomize participants to study groups after determination of eligibility, but before baseline testing. Conversely, other major studies do not randomize participants until all baseline testing is completed. We decided to determine initial eligibility by a telephone screen, an orientation session, and a preliminary screening examination prior to

the run-in period. We decided to determine final eligibility and conduct randomization after the run-in because 1) the baseline examinations may reveal that some participants meet exclusion criteria not discovered at the preliminary screening; 2) the screening examination will include blood chemistries, and more complete laboratory data previously not available; and, perhaps most importantly, 3) we wanted to use the baseline examinations as part of the run-in to improve adherence rates.

Dependent Variables. HbA1C concentration is the primary dependent variable and the variable used in testing the primary hypotheses. Other outcomes of interest include HOMA index, resting blood pressure, CRP, total body fat and muscle mass, cardiorespiratory fitness, muscular strength, and metabolic measures including serum cholesterol and triglyceride. We include them here as outcome variables, but we recognize that they are sometimes independent or control variables in other analyses. All outcomes will be assessed at baseline and after 9 months. HbA1C will also be measured monthly.

Blood Draws and Measurement of HbA1C (Primary Outcome). Subjects will be asked to fast at least 10 hours prior to blood draw. In addition, subjects will be asked to refrain from consuming alcohol or participating in an exercise training session at least 24 hours prior to blood draws. Trained personnel (nurse, phlebotomist, or lab technician) will draw the blood from an antecubital site on either arm into vacutainer tubes prior to 9:30 am. Standard OSHA guidelines will be followed at all times. At the baseline and 9-month assessments, approximately 20 ml of blood will be drawn. Of this, ~10 ml will be immediately sent to the laboratory for HbA1C, lipid assessment, and blood chemistries. The remaining 10-ml sample will be centrifuged under refrigerated conditions and the supernatant will be extracted and divided into 1-ml portions. Vials of plasma (3-4), serum (2-3), and red blood cells (2-3) will be stored at −80°C for future analyses. In addition, monthly HbA1C testing will be conducted via finger prick and Bayer DCA2000+ HbA1c analyzer.

Homeostasis Model Assessment (HOMA) Method. Homeostasis Assessment Model is a mathematical model that can be used to quantify insulin resistance (expressed as a percentage of normal).[73] The insulin resistance index assessed by HOMA will be calculated as follows: HOMA = (FPG * FPI) / 22.5, where FPG is fasting plasma glucose level (mmol/l) and FPI is fasting plasma insulin level (µU/ml).

Resting Blood Pressure. Blood pressure will be measured at rest, under controlled conditions. At least 2 resting blood pressure measurements will be obtained after a 5-min rest, with the subject in a seated position in a quiet room. The average of the 2 values will be used.

Total Body Composition by DEXA.
Dual X-ray absorptiometry (DEXA) scans will be performed using the Hologics QDR 4500A whole-body scanner during an outpatient visit. The protocol requires that participants lie on a table wearing a hospital gown and no metal containing objects, while the scanner emits low energy X-rays and a detector passes along the body. The scan takes 4-6 minutes and the radiation dose is less than 1 mrem, equal to about 12 hours of background radiation. Two distinct energies are used to determine body mineral and soft tissue content. An attenuation ratio is determined from a known tissue content. Variations of the attenuation ratio determine the fat content of the tissue at each pixel thereby calculating the percentage body fat. The scans are analyzed with the latest software QDR for Windows V11.1. The Hologics QDR 4500A scanner has a weight limit of 300 lbs; therefore, participants weighing over 300 lbs. will not have this procedure done.

Muscle strength and endurance will be measured via isokinetic testing on a Cybex machine during knee extension and flexion. Isokinetic testing allows for measurement of force velocity or

power velocity curves as well as peak force and peak power. Unlike other methods of measuring strength, isokinetic testing is quick and less likely to cause injury in subjects not used to performing this type of testing. The procedure involves an initial test of 5 repetitions at 60 degrees/sec. to measure peak force and power followed by a test to fatigue at 180 degrees/sec.

Measurement of CRP.

Because aspirin or anti-inflammatory medications may modify CRP levels, participants will be asked to refrain from using these types of medications for 48 hours prior to the blood draw. If the participants take aspirin or other anti-inflammatory agents on a daily basis, perhaps on physician's advice for prevention of CHD or for other existing medical problems, and they were taking it at the time of screening, they will not be asked to abstain for testing purposes. Use of anti-inflammatory agents will be recorded and can be used in analyses for adjustment or for exclusion of participants for selected analyses. CRP is measured by the high sensitivity assay on a Prospect nephelometer (Dade Division of Baxter Healthcare Corporation, Delaware). We understand that there may be other important markers (for example, IL-1, IL-4, IL-6, and IL-10), which we will not measure, but the frozen blood samples allow for potential future analysis.

Cardiorespiratory Fitness Testing will occur following a medical history review and examination. All exercise testing will be conducted using a treadmill. The exercise test will be completed at months 0 and 9. Prior to this test, a resting ECG will be taken and resting HR and blood pressure will be measured. The maximal exercise test will be completed at the baseline examination and 9 month follow-up using a modified Balke protocol. Several measures suggestive of cardiorespiratory improvement will be examined during each test inclusive of VE, VO_2, VCO_2,, and RER and will be measured continuously throughout the exercise tests using a Parvomedics True Max 2400 Metabolic Measurement Cart (Salt Lake City, UT). Following each exercise test, we will determine maximal heart rate, maximal oxygen uptake (VO_2), pulmonary ventilation (VE), ventilatory equivalents for oxygen (VE/VO_2), carbon dioxide (VE/CO_2), end-tidal partial pressure of oxygen ($PETO_2$), and carbon dioxide ($PETCO_2$). Ratings of perceived exertion (RPE) will be obtained using the Borg scale. The same maximal exercise test protocol will be used at the baseline and 9-month evaluations. The same gas exchange and cardiovascular variables will be measured.

Psychosocial Measures or Mood and Quality of Life Measures

The CDC Health-Related Quality of Life 14-Item questionnaire and the SF-36 questionnaire will be administered at baseline and follow-up to assess the impact of AT, RT and AT+RT exercise on quality of life in individuals with type 2 diabetes.

Covariables

Other Laboratory Measurements.

We will draw blood for a complete blood count, lipids, and a chemistry panel (including glucose) to help rule out occult medical issues at baseline. These measures will also be repeated at 9-month follow-up visit, as this too will be a valuable source of outcome data.

Monitoring Daily Activity and Weight.

We will monitor routine daily energy expenditure in all participants by obtaining objective data on physical activity with step counters throughout the study period, and these data can be used as a covariate in data analyses. It has been suggested that individuals who start a new exercise program may become more sedentary during the rest of the day. On the other hand, one resistance training study found individuals randomized to resistance exercise greatly increased lifestyle physical activity.[74] The monitoring planned for HART-D will allow us to evaluate the issue of changes in other physical activities and adjust if necessary. We will measure weight once a week prior to the first exercise session of each week. This weight is immediately entered in the Vital Link system and used to fine-tune the exercise prescriptions.

Monitoring Blood Sugars.

We will ask participants to provide the highest and lowest blood sugar levels measured during the days since the last exercise session. We will establish both high and low alert values, which will trigger an e-mail to be sent to the study Certified Diabetes Educator (CDE). This value will also allow the exercise intervention staff to evaluate if exercise is safe for the participant at that time. The CDE will contact the participant, either immediately or prior to the next exercise session, to discuss the need to see their primary doctor or other strategies to avoid abnormal blood glucose values. Blood glucose levels may be tested prior to and following exercise sessions. Participants with blood glucose levels < 100 mg/dL will be given a small snack, and asked to wait 20 minutes before retesting blood glucose values and receiving clearance to exercise. Participants with blood glucose values above 250 mg/dL will undergo a urine ketone test. If urine ketones are present or blood glucose levels are above 400 mg/dL, the CDE will be notified and the participant will be sent home.

Monitoring Medication Changes and Compliance.

Participants will be asked to bring all their medications to a run-in appointment. A trained staff member will carefully transcribe the name and dose of each medication. At each exercise training session, using the Vital Link System, participants will be asked about frequency of medication use and changes in medication doses or types since the last exercise session. If participants note there has been a medication change the computer will ask for specifics. Changes in doses of individual medication as well as total number of medications will be evaluated over the course of the study and at the final examination. The CDE will also discuss medication changes during monthly visits.

Monitoring Dietary Changes.

We believe that it is important to account for potential changes in dietary habits, and thus we will obtain food frequency questionnaires at baseline and the 9-month follow-up. This will provide us with the ability to adjust the analysis for changes in dietary habits if necessary.

Anthropometry.

Height and weight will be measured using a standard stadiometer and balance beam scale. Measurements will be taken without shoes and recorded to the nearest centimeter and 0.1 kg. Waist circumference will also be measured to the nearest millimeter.

Independent Variables.

Secondary outcome variables are either dependent or independent variables, depending on the specific analysis. However the primary independent variable for the study is treatment group. Here we describe the exercise treatments and relevant factors in its selection and description.

Rationale for Selection of Exercise Intervention.

As the majority of T2D treatment guidelines recommend weight loss, we had many discussions related to evaluating a weight loss program compared to an exercise program alone. We elected not to test a weight loss intervention for a variety of reasons. There are a number of studies that have shown weight loss to be beneficial to T2D. However, these studies cannot address the source of the improvement. Is it the negative caloric balance, better quality diet, increases in physical activity, or a combination of these or other factors? Further and most importantly, many individuals with abnormal glucose/insulin metabolism are not overweight or obese and thus these individuals need treatment options other than weight loss. Exercise may be one such option, and as detailed above, is deserving of further investigation as such.

Rationale for Selecting Types of Exercise Intervention.

Exercise is typically characterized by its type, intensity, and session duration and frequency. The two most commonly used types of exercise are aerobic (endurance) exercise and resistance (strength) exercise.[75] The main effect of aerobic endurance training is an increase in ability of

the cardiorespiratory system to deliver oxygen as well as an increase in the oxidative capacity of skeletal muscle, which generally improves cardiorespiratory fitness. Resistance exercise has been shown to be a potent stimulus to increase fat free mass, muscular strength, and muscular power. Intervention studies have demonstrated that many positive effects on insulin sensitivity and glucose homeostasis occur in response to aerobic training. Studies examining the effects of strength training have found similar improvements in insulin resistance.[76 77-79]

Although aerobic and resistance training both improve insulin sensitivity and thus glucose utilization, they largely do so through different physiological mechanisms. In general terms, aerobic training improves muscle physiology (without changes in muscle size) through increases in GLUT4, skeletal muscle capillarization, mitochondrial size and density, and glycogen synthase activity while resistance training increases to total muscle mass and strength with only minor changes to muscle physiology. Given that aerobic and resistance training appear to improve glucose metabolism through different primary mechanisms it is not unreasonable to hypothesize that a combined aerobic and resistance training will improve glucose metabolism more than aerobic or resistance training alone. There is preliminary evidence to support this hypothesis and there is a need for a definitive trial examining the role of resistance training, alone and in combination with aerobic training, in lowering HbA1C in individuals with T2D.

Study Schedule

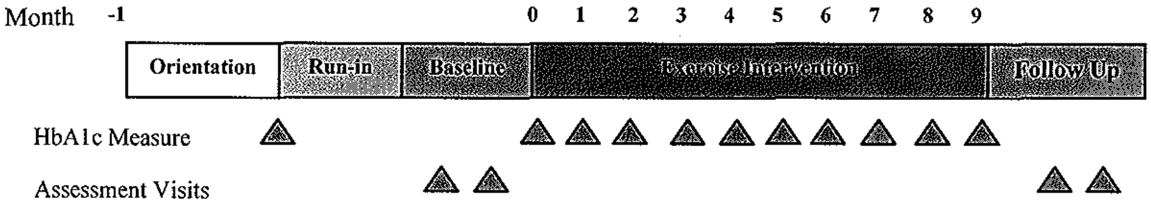

Assessment Visits include:
Anthropometrics, Blood draw, DEXA, Fitness Testing, and Strength Testing

Rationale for Selection of Exercise Doses.

Rationale for Selection of Aerobic Exercise Dose.

The recommendation that individuals with T2D obtain 30 minutes of moderate intensity physical activity on most days represents a consensus of the recent reports.[80, 81] We used this recommendation as the basis for determining the specific aerobic exercise doses to be utilized in HART-D,[82] that is, 30 minutes of moderate intensity exercise for 5 days of the week, which will produce an exercise dose of 12 KKW. This dose of aerobic exercise for an 84-kg person would mean this person would have to expend 1008 kilocalories per week. However, we are concerned about an excessive subject burden if we asked them to come to the supervised exercise sessions for 5 days a week, and that this burden might have an adverse effect on adherence. Furthermore, data from studies on frequency of exercise sessions show little difference in physiological changes for exercise frequencies of 3 or more days per week, provided the total weekly exercise dose is held constant. Therefore, the target in this study is for participants to obtain their weekly exercise dose in 3 or 4 sessions. In 3 sessions this would be 336 kcal expenditure per exercise session and in 4 sessions this would be 252 kcal per exercise session. Based on our experience in previous clinical trials, where the average participant is unfit (VO_2 max of 15 ml/kg/min), these exercise doses are feasible and well tolerated.

We also considered evaluating different exercise intensities (high versus low) in the study, but decided on an intensity range of 50%-80% of VO_2 max. The various public health recommendations for physical activity recommend moderate intensity exercise for sedentary individuals. It also seems likely that the moderate intensity we selected will be associated with better adherence than if we used higher intensities. There are data to show that exercise at 50%

intensity is sufficient to produce clinically significant physiological adaptations in sedentary individuals.[82-88]

In summary, many factors must be considered when defining an aerobic exercise dose. Physical activity recommendations to the public have recently been presented in minutes per day [82, 83] or kcal per day.[85] There are problems in using these approaches in a highly controlled efficacy exercise study such as HART-D. Differences in rate and amount of improvement in fitness and weight loss illustrate 2 potential confounding problems. Therefore, we decided to use the consensus public health recommendations to define an original exercise dose in KKW. We think this approach is the option that offers greatest control of confounding by such issues as discussed above, and fewer disadvantages than other options.

Stretching was chosen as the exercise modality of for the control group, but there is little evidence to suggest that mild stretching will have any measurable effect on HbA1C levels in any population. Furthermore, the group will benefit from continued interaction with HART-D staff and fellow participants, and we feel that this interaction will also improve adherence rates in this group.

Rationale for Resistance Doses for Both Resistance Alone and Combined Groups.

For the resistance-training program, there were several considerations to be taken into account before formulating the final exercise prescription for the AT+RT group and the RT group. For the AT+RT group, the development of the proposed resistance-training schedule was straightforward and based on current recommendation for T2D populations.[89, 90] Both the ACSM and the ADA suggest that, in addition to an aerobic exercise, individuals with T2D should participate in 20 minutes of resistance training twice a week. Each resistance workout should consist of 1 set of 8 to 10 exercises with each set consisting of 10-12 repetitions at an intensity of 40%-80% of 1-RM. Because the recommendations are well described and we are testing the effect of the current recommendations on HbA1C, there was little room left for consideration of other resistance regimens.

In selecting the exercise dose for the RT group, we had to choose between the resistance training protocols from current exercise guidelines for patients with T2D (described above) or the more demanding protocols from the most successful resistance training and HbA1C studies. In general, the more demanding resistance training protocols consist of 30-45 minutes, 3 days a week. The repetitions of each exercise and the relative intensity are the same as described in the above paragraph, but the number of sets and number of exercises are greater.

To provide uniformity of exercise and efficient use of space, we chose a circuit protocol. Past studies have shown that physiologic benefits are maximized when resistance training focuses on large muscle groups.[91, 92] Therefore, the primary resistance training exercises use large muscle groups in a functionally relevant motion. The primary exercises used in this study are similar to those used in most previous resistance training research in individuals with T2D.

Length of Trial.

We considered 6, 9, and 12 months as trial lengths. Given that our primary outcome is HbA1C which is a measure of glucose control over the previous 8 to 12 weeks, we felt this study warranted a longer study period. 6 months might be too short to see the maximal benefits from resistance training and that 12 months would be challenging to keep participants in the study, which requires 3-4 weekly sessions in our exercise laboratory.

Exercise Protocol.

All training sessions will occur in The PBRC Fitness Center. The machines in the center have adjustable seats and pads to accommodate a variety of different body shapes and sizes to promote proper lifting biomechanics. The facility has a real time touch screen computer system, which is used to track the progression of the participants both within a given session, week, and during the entire study. This system, Vital Link, is described in greater detail in the Data

Management section, and as noted above this system will be used to track changes in weight, medications, and physical activity, and monitor self-report glucose values. The exercise laboratory will be open from 6:30 am until 7:00 pm, Monday through Friday, to allow maximal scheduling convenience. Further, there are large locker rooms equipped with lockers and showers available for the use of study participants.

Stretching and Relaxation Protocol.

One or more times a week, participants in the stretching group will report to Pennington Biomedical Research Center to complete a 45 minute exercise session consisting of light stretching and relaxation exercises. All exercise will be guided and supervised by a trained professional.

Aerobic Training Protocol.

The target training intensity will be self-selected between 50% and 80% of VO_2 max and the frequency will be 3 or 4 times per week. The number of days per week will be individualized for each participant, based on initial fitness level, travel, and other scheduling issues and personal factors. Participant weight will be used to determine weekly energy expenditure in kcal to achieve the target of 12 KKW, and the frequency of sessions will be determined to establish a manageable time goal, 3 or 4 sessions per week. The weekly caloric expenditure may be distributed over 5 days, initially, if a participant is unable to complete the prescribed dose in 3 or 4 sessions. Participants will exercise on a treadmill (or cycle ergometer, if necessary).

A computer-controlled exercise training management system will be implemented. The system allows for input of relevant data for each participant (study ID #, group assignment, VO_2 max, resting and maximal heart rate, body weight, and whether the participant is on a 3- or 4-day/week exercise schedule). The participant will select his or her preferred exercise intensity within 50%-80% intensity range to determine speed and grade for the treadmill, and the computer will also indicate the number of minutes the participant is expected to exercise to reach his or her exercise dose in KKW. The training program will consist of a 5-min warm-up at a progressively increasing PO until the prescribed training intensity is reached. The duration of each session will be the time required to reach the target weekly caloric expenditure divided by exercise frequency (e.g., 3 or 4 times per week). During exercise we will monitor heart rate by a Polar transmitter (Finland). With the PO on the cycle ergometer (or grade and speed on the treadmill), the total kcal expended each minute will be calculated, and the number of minutes needed to reach the target energy expenditure will be indicated. Participants will be allowed to read, listen to music, or watch television while they exercise.

Because all participants may not be immediately capable of exercising at their required dose, there will be a progression to the 12 KKW dose. This gradual increase in total energy expenditure is expected to minimize fatigue, soreness, injuries, and dropouts. By the end of the 1st month, all participants should be able to perform 100% of the dose over 3 to 4 days; however, for a small percentage of participants, 5 days of exercise may still be needed. We believe allowing this flexibility will enhance adherence and reduce dropout rate.

The estimated number of minutes per week required by a participant in the AT group to expend the required 12 KKW depends on actual speed and grade while walking. For example, a 70-kg participant will need to achieve an energy expenditure of 840 kcal/wk. The required number of minutes per walking (e.g., speed 4 mph, 2% grade) session with the frequency 3, 4, or 5 days/wk corresponds to 45, 34, and 27 minutes, respectively.

Resistance Training Only Group Protocol.

Prior to each session there will be 5 minutes of light exercise as a warm-up (chair stands, short walk, etc). The strength training will be performed on 3 days each week. Participants will do 2 sets of 8-12 repetitions on each exercise, and a third set of leg exercises will be performed. We estimate it will take participants approximately 45 minutes to complete a resistance training session. Participants will start strength training at a low relative intensity. Each consecutive week the load will be increased, as tolerated, until the weight lifted is equivalent to

10RM, or the amount of weight a participant is able to lift 10 times for a given exercise. This is equivalent to roughly 80% 1RM. The ramping protocol will help to acclimate participants to resistance training and may decrease injuries, dropouts, and other adverse events. Resistance will be increased when an individual can comfortably perform 12 repetitions of the exercise during 2 consecutive exercise sessions. At the end of each session there will be a 5-minute cool-down period (flexibility and stretching), resulting in the total time of the entire resistance training session being approximately 55-60 minutes.

Combination Training Group Resistance Training Protocol.

Participants in the AT+RT group will expend 10 KKW as part of the aerobic training regimen as well as a less demanding resistance program than the RT group. We considered having the combination-training group perform exactly the same RT protocol as is planned for the RT group. However the time required for this would be too long, and we felt that this might adversely affect adherence to the exercise regimen. Therefore we slightly reduced the RT component of the combined protocol. In accordance with the current ACSM and ADA guidelines for individuals with T2D, participants will complete 10 to 12 repetitions of each exercise at 40%-80% of 1-RM. The ramping protocol in the AT+RT group will be identical to that in the RT-only group. We anticipate it will take participants approximately 20 minutes to complete the resistance training session. Resistance will be increased when an individual can comfortably perform 12 repetitions of the exercise during 2 consecutive exercise sessions. During the days that individuals choose to perform both aerobic training and resistance training in the same session, we anticipate the total time spent exercising to be 55 minutes to 60 minutes.

Exercise Safety Considerations in a Diabetic Population.

The study CDE will be responsible for training both staff and participants about glucose control and exercise in individuals with T2D. For the staff, the physician will provide ongoing education and serve as a consultant for helping deal with glucose monitoring before and after exercise training sessions. For the participants, as part of the run-in, presentations will be provided on what to expect in terms of glucose control-associated problems with starting a new exercise program. The CDE will be responsible for reviewing medication records as well as daily glucose monitoring records that are collected via Vita Link. The CDE will be expected to address any mediation concerns with participants when needed and bring important issues to the attention of the participant's physician. During monthly visits with participants, the CDE will review past blood glucose levels with participants, discuss medication changes, provide lifestyle modification guidance, and refer participants to their primary care physician, when warranted.

We appreciate that when exercising a high-risk population such as individuals with T2D there are additional issues that must be considered to assure participant safety. There are a number of excellent summary articles that address potential health risks related to T2D and exercise training. [93, 94] Hypoglycemia can become an issue with individuals with T2D exercising more than an hour. Severe hypoglycemia is defined as any episode of loss of consciousness/seizure or documented hypoglycemia (glucose <70 mg/dL or 3.9 mmol/L) that prevents self-treatment, or requires hospitalization or treatment by emergency personnel. Minor hypoglycemia is defined as self-reported transient symptoms such as lightheadedness, tremor, shaking, sweating, tingling, blurry vision, trouble concentrating, etc., that are self-treated by ingestion of carbohydrates and resolved on their own or within 15 minutes of such self-treatment. Throughout the study, participants who are taking sulfonylureas, repaglinide, or nateglinide may need reductions in T2D medication(s) to reduce their risk of hypoglycemia. As described above, at each exercise session when participants sign-in they will enter their lowest and highest self-monitored glucose levels since the last session into the Vital Link system. Glucose <70 mg/dL will trigger an e-mail to the CDE, who will follow-up with the participant. The HART-D staff, primarily the CDE, will notify the participant's primary care physician (PCP) that changes in diabetic medications may need to be made to prevent hypoglycemia. Medication reduction for participants will be done only by the participants' PCP.

All study participants will receive education on the risk of hypoglycemia prior to randomization. There will be blood glucose monitoring devices available in the exercise lab for participants to have their glucose levels checked before and after exercise. Participants will be encouraged to review this information with their PCP and with the study staff. Participants will also have access to water, snack food, and juices with 6-8% carbohydrate solutions.

Peripheral neuropathy is another concern when training individuals with T2D. We will have an examination room available where participants can inspect their lower legs and feet after each exercise session. Further, during the run-in period participants will be counseled as to proper exercise footwear and strategies to avoid blisters/pressure sores. Nephropathy is common in T2D and there is no evidence showing a deterioration of renal function with aerobic or resistance training. Retinopathy is also common in T2D and rises in system and retinal blood pressures with resistance training have been suggested as a potential concern. To our knowledge, there is no scientific literature showing acceleration in retinopathy with chronic exercise and interocular pressure is actually reduced with chronic aerobic training. Nonetheless, participants will be reminded to breathe freely during the resistance training and avoid Valsalva maneuvers. It should be noted that though special risks are present when individuals with T2D exercise, for most individuals the benefits of regular exercise far outweigh the risks.

In summary, having a run-in period that is focused on safety strategies for individuals with T2D starting an exercise program combined with the supervising physician and CDE who are focused on participant safety will help reduce the likelihood of exercise-related adverse events in this trial.

Interventions for All Participants

All participants will attend an approximately 60 minute individual session with a CDE and will receive written information regarding standard lifestyle recommendations for individuals with type II diabetes. During this session, participants will be encouraged to follow Food Pyramid Guidelines, consume National Cholesterol Education Program step I diets, lose 5-10% of their initial body weight, gradually increase physical activity with a goal of at least 30 minutes of exercise 5 days per week, avoid excessive alcohol consumption and stop smoking, if applicable. Because participants are not randomized until after the completion of all baseline assessments, the participants randomized to the control group will also complete the educational run-ins following orientation. The educational run-ins are comprised of PowerPoint presentations on various topics, including, healthy eating habits, importance of physical activity, proper foot care for persons with diabetes, how to prevent hypoglycemia, and other basic diabetes management information.

Participants in all groups will be required to maintain relationships with their primary care physicians. We will not, in any way, restrict alterations to medication use prescribed by the participants' primary care physicians. We will simply record such changes, and this data will be used upon study completion to adjust for changes in glucose control resulting from modifications in medication use for all groups.

Medical Management by Participants' Own Health Care Providers.

The HART-D trial is neither designed nor staffed to provide comprehensive medical care to all participants, nor is this necessary to address the principal study objective of the trial. HART-D participants will receive their diabetes and general health care from providers outside of the study. This approach may maximize the willingness of physicians to refer patients to the proposed study.

Participants in HART-D will need ongoing medical care during the duration of this trial because they have T2D and will need to receive medical care for the management of this

disorder. Additionally, many participants will have hypertension and other cardiovascular risk factors, such as dyslipedimia, that require medical management. Furthermore, although some types of pre-existing illness will exclude potential volunteers from participation, a wide range of medical illnesses will not be cause for exclusion from participation in the proposed study, and yet will require ongoing medical care. It will be the policy of the HART-D that all participants should have a source of medical care independent of the HART-D staff. If a participant does not have an identifiable source of medical care, enrollment in HART-D will be deferred until a health care provider is established for the participant. Ultimately, this is the responsibility of the participant. Our staff will assist the participant by referral to a health care facility. It is unlikely that participants will, for a variety of reasons, lose contact with their current physicians during the course of the study. If participants change medical care, medical information will be sent to the new physician. We will ask our participants to provide information about prescriptions, adjusting medication doses, blood pressure checks, and other treatments.

Adherence to Exercise, Dropouts, and Participant Incentives.

While ethical considerations require that participants be allowed to withdraw at any time, every possible research-based strategy, including financial incentives, will be used to minimize dropouts and to maximize the number of individuals completing the testing and treatments.[95-97] This will involve organizing the recruitment, testing, and training programs to enhance adherence. We have learned a great deal in this regard from our past clinical trials in terms of the importance of creating a pleasant research environment and friendly and welcoming staff. In addition, we are very clear from the orientation session onward about what we expect from participants and what they can expect from us. We believe that keeping participants very well informed enhances adherence to the intervention and test schedules. We are assuming equal adherence for all study groups because we have no data to predict otherwise, and this has been achieved in our previous studies. The strategies described here will be applied to all participants, and we will use individual problem-solving approaches where necessary to maximize adherence. Staff will be trained to develop rapport with the participant by communicating expectations of the participant's role and the staff person's role. We have found that personalizing this relationship as much as possible in a professional context enables participants to be more direct about problems.

Adherence to Exercise.

Study results could be adversely affected by poor adherence to the exercise protocol. This could jeopardize the internal validity (e.g., differential attrition across treatment groups), the generalizability of the findings, or the ability to determine treatment effects. We believe there are 3 major areas where poor adherence to the protocol is a major issue. First, the participant may decide to exercise more or less than their group assignment. Briefly, exercise sessions will be completed in the laboratory where individuals will be closely monitored. In addition, the importance of performing the prescribed exercise dose will be reinforced at every opportunity. A second concern is that some individuals may fail to complete their supervised exercise sessions. Prior to randomization, study staff will share expectations regarding attendance for 3 to 5 sessions per week and will ask participants about their expectations regarding randomization. Participants will be asked during the pre-randomization visits whether they can make this type of commitment. If they reply that they cannot complete exercise sessions regularly and accept randomization, they will be excluded from the study. If they say yes, we will ask them to sign a behavioral contract that affirms their commitment to complete exercise sessions. There is also the possibility that individuals will say they can meet the time commitment, sign the behavioral contract, and then not be able to attend their scheduled appointment. Participants will be asked to call as far as possible ahead of time to

reschedule appointments. The exercise laboratory will be open 5 days per week for 12 hours/day to accommodate a variety of schedules. Third, participants may get sick or injured, their children, parents, spouse, or others may become ill and require care, or some may travel for work or vacation. Therefore we are defining acceptable adherence to the study protocol as completing 90% or more of the total amount of exercise prescribed. This 90% criterion allows an individual to miss a total of 3.6 weeks or a maximum of 14 sessions of the total required. This provides time for vacations, illness, and other family and work commitments. We have become very adept at adjusting schedules to help individuals prepare for missed exercise sessions for personal reasons such as vacation or work travel. In addition to the procedures described above that will provide flexibility, financial incentives will also help ensure adherence. We believe good adherence will be achieved by these procedures and by clearly defining expectations within a supportive atmosphere that enables staff and participants to solve the logistical problems that inevitably arise. As described above, participating in 90% of the planned sessions will be labeled as successful adherence, which should be a very obtainable goal based on our current and past experiences. We expect that we will be able to produce similar results in the proposed study.

Dropouts.

Some participants may drop out (defined as failing to return for the 9-month examination). Our power calculations allow for a dropout rate of 15%, and we believe the run-in will exclude many at high risk of dropout. Personalization and problem solving approaches described above to ensure adherence to exercise will also be helpful in preventing dropout. We feel that the provision of complete information concerning the amount of time and effort required of participants, the run-in period, and a sizable financial incentive will be sufficient to maintain a drop-out rate far below 15%. Our power calculations are based on intention-to-treat analysis with dropouts and are covered in below.

Participant Incentives.

We will provide $200 per individual as an incentive for participation in the study. We realize that this is a substantial amount, but we think that this is appropriate because our objective is to evaluate the effects of exercise on HbAlC, and excellent adherence to both intervention and measurement is necessary. If we were testing the effectiveness of an exercise intervention as a public health strategy, such a high payment for participation in the intervention by exercisers would not be appropriate. We are not testing whether or not financial incentives encourage individuals to exercise, but are evaluating specific responses to various exercise regiments.

The payment will be reduced by $15 for every 10% drop in compliance beyond the 90% adherence target in the exercising groups. There will also be a $10 reduction in compensation for every missed CDE appointment. The maximum deduction for failing to exercise/complete monthly visits will be $100. Thus a person who does not come to exercise/monthly sessions at all will still receive $50 for completing the baseline assessments and another $50 for completing the 9-month evaluation. Participants in the SC group will be eligible to attain the $200 if all monthly visits with the CDE and monthly questionnaires are completed. This incentive payment schedule is designed to promote both the adherence to exercise program and to complete the follow-up examinations.

Risks and Benefits:

Procedure:	Risk:
Fasting blood samples	Participants will undergo needle sticks during visits where blood samples are collected. Risks include pain, lightheadedness, infection, bleeding or bruising at the site of injection; however, the staff will use proper technique while taking blood samples in order to reduce the risk for these unwanted effects.
Body composition assessment by DEXA	Minimal x-ray exposure. Example: 12 hours background radiation from the sun. Exposure to radiation can harm an unborn child and pregnant women are not allowed to undergo this procedure. All women will complete a pregnancy test prior to each scan.
Maximal Exercise test	The aerobic capacity test will cause a temporary shortness of breath identical to that experienced following heavy exercise, and may lead to mild muscle soreness the next day. Potential side effects of aerobic capacity testing include dizziness, fainting, blood pressure elevation, heart arrhythmia (heart beat irregularity), and heart attack, but the risk of these events is very low, and a doctor or nurse practitioner will be present during all treadmill testing.
Exercise training	Exercise that is not commonplace or routine may elicit muscle soreness and stiffness. This is normal at the beginning of an exercise program and should subside with time. To minimize this, the exercise will commence at a low to moderate level and will be increased gradually, once every 4 weeks. All exercise sessions will be supervised by an experienced exercise physiologist or personal trainer. Warm-up and cool-down stretches will be provided and recommended at each training visit. Blisters and overuse injuries are also possible. If prolonged soreness or stiffness occurs, or more than slight swelling occurs, participants are advised to notify the Principal Investigator or Medical Investigator. With any physical activity, there is a chance of muscle injury, ligament and tendon injury, as well as skeletal injury.
Muscle Strength & Endurance testing	As with any new activity, participants could experience some soreness in the muscles tested on the following day.
Electrocardiogram (EKG)	There is a small possibility there may be some redness or itching if allergic to the electrodes' adhesive. In rare cases, the adhesive has caused skin discoloration at the site of the electrodes.

Participation in the screening process of this study may cause all or some of the side effects listed above. In addition, there is always the risk of developing previously unknown side effects. The investigator is willing to discuss any questions you might have about the severity, frequency and the duration of these risks and discomforts.

Risk Classification: The study risk cannot be classified as minimal.

Minimizing Risks: Every effort will be made to minimize all foreseeable risks associated with participation in this study. Continuous monitoring by the PI and/or the medical investigator will minimize all potential risks and discomforts.

Potential Benefits: Participants will receive some information about their health and will be allowed to request that the results of any test be shared with their primary care physician. The cost of the blood tests, body composition measures, exercise testing and 9 months of supervised exercise or personal training would be well over $1000 if obtained from a private clinic or doctor's office. Also as a result of the exercise training participants may experience an increase in fitness level and some changes in body composition such as a reduction in body weight and a loss of body fat.

Financial Obligations of the Subjects: None

Emergency Care and Compensation for Research-Related Injury:
No form of compensation for medical treatment is available from the Pennington Biomedical Research Center. In the event of injury or medical illness resulting from the research procedures the research volunteer will be referred to a treatment facility. The Pennington Biomedical Research Center is a research facility and provides medical treatment only as part of research protocols. Should a volunteer require medical treatments, community physicians and hospitals must provide them to him/her.

Data Management and Analyses

Data Management. Exercise test data will be downloaded from the metabolic cart directly following each test and reviewed for integrity. Data from the exercise-training laboratory will be captured with the Vital Link system and appended to the study database weekly. Vital Link is real time touch screen computer interface that is custom designed and programmed for exercise session monitoring. The Vital Link system is designed to minimize data entry errors through the use of standardized pre-coded forms for each participant and assuring all values entered fall within an acceptable range. Further, Vital Link eliminates the need of scanning exercise intervention forms and the verification and data cleaning associated with this process. All data will be incrementally backed up each day, and each week a full backup will be performed

Data Analysis.

All data analyses will be implemented with the SAS/STAT® v9 software. All variables will be tabulated and inspected for completeness, range, and internal consistency, and will be edited as necessary. Baseline values of all variables will be tabulated by treatment group to identify imbalances and potential confounders. Primary statistical analyses of primary and secondary outcomes will evaluate treatment effectiveness and will include all participants who complete assessments at 9 months, regardless of adherence to protocol, with patients grouped as randomized. No incomplete data will be imputed or simulated in the primary analyses. Ancillary analyses will then examine treatment efficacy in the subgroup of adherent participants; evaluate treatment mediators such as changes in diet, medication, and weight during treatment; evaluate treatment moderators such as pre-treatment body habitus; assess the impact of incomplete data due to attrition; and examine treatment response at 3 and 6 months. Statistical analyses will generally be based on pre- to post-intervention change-score ANCOVA models for treatment differences in mean response, with adjustment for baseline levels and important covariates. Results will be reported as least-squares adjusted means and associated 95% confidence intervals. With a moderately large sample size and nearly equal numbers per treatment group, ANCOVA should be robust to non-normality. However, we may consider non-parametric (e.g., Wilcoxon signed-rank) tests or transformation of secondary outcome measures. Regression diagnostics will be used to assess model fit and to identify and examine outlying, influential, and ill-fitting data points.

Primary Outcomes:

The primary outcome measure is percent HbA1C, each month post randomization. No interim analyses are planned. Out of concern for Type I error rates in performing repeated hypothesis tests, we have pre-specified only one primary outcome measure with two specific aims. Subgroup analyses (e.g., by age, gender, insulin use, ethnicity or BMI) for treatment-effect variations will be interpreted with caution and formally tested for interaction. Other outcomes are secondary and exploratory, and results will be interpreted with appropriate caution.

Primary hypothesis 1: that at 9 months, the AT+RT group improves HbA1C more than AT alone and more than RT alone, and all three exercising groups will improve more than the standard care group. Covariates will include baseline HbA1C and may include age, gender, BMI; medication use (such as insulin and hypoglycemic agents) and dietary intake (macronutrients and energy) at baseline, and prior medical history.

Primary hypothesis 2: that at 9 months, AT alone and RT alone improve HbA1C equally. We will use ANCOVA to compare mean change scores of AT vs. RT, with covariates as outlined above.

Secondary Outcomes: Secondary outcome measures at 0 and 9 months include HOMA index, resting blood pressure, CRP, total body fat and lean muscle mass, cardiorespiratory fitness, muscular strength, quality of life, medication changes, and metabolic measures including serum cholesterol and triglyceride.

Secondary hypotheses: We will evaluate the effects of standard care, AT alone, RT alone, and AT+RT on the secondary outcomes at 9 months. Analyses will be based on change-score ANCOVA models as outlined above. Transformation of the data toward normality (e.g., using logarithmic or Box-Cox transforms) will be considered.

Efficacy analysis: Ancillary analyses of all primary and secondary hypotheses outlined above will also be conducted on the subset of compliant participants, defined as those who complete 90% of weekly exercise sessions to protocol. Treatment comparisons of participants who achieve pre-specified increases in fitness or strength will also be of interest.

Mediators analysis: Mediators are post-baseline variables that come between treatment and outcome, are correlated with treatment group, are predictive of the outcome measure, and explain some or all of the overall treatment effect on the outcome measure. A potential mediator will be added to the foregoing ANCOVA models as an independent effect. Of particular interest are changes in weight, diet, medication, and glucose monitoring, and adherence to treatment. For example, a time-weighted measure of cumulative weight loss can be calculated as the product of baseline weight times 9 months, less area under the weekly weight profile curve from 0 to 9 months. Entered as an independent effect, this measure may explain some of the differences between treatment arms in changes in HbA1C. Type of medication (e.g., hypoglycemic) will be categorical, with amount figured as adherence times regimen times dose. Time-weighted cumulative change in dietary intake of carbohydrate, fat, and protein as a percentage of total energy will be examined as mediators. Percent of exercise sessions completed will also be examined as a mediator.

Moderators analysis: Moderators are baseline variables that alter the effect of treatment on the outcome measure. Of interest are age, gender, body fatness and weight, medical history, and type and dosage of medication. Informally, moderators can be categorized in subgroups (e.g., age groups) and treatment effects compared across subgroups. More formally, moderators will be entered in the ANCOVA models as interaction terms to test for differences in treatment effects across moderator levels. We anticipate that power will be limited for such analyses.

Incomplete data: As in most if not all trials, there will be noncompliance, dropouts, and missed visits. The reasons for dropout and noncompliance may be related to the outcome (e.g., lack of improvement or deterioration of symptoms), to treatment (e.g., injury or exercise intolerance), or to initial severity of illness. Although we anticipate relatively low attrition and noncompliance based on our experience in previous and ongoing trials, treatment- and outcome-related dropout and differential attrition could bias the treatment comparisons. These complications always pose difficult problems for data analysis and interpretation of findings. To investigate dropout rates after trial entry, the log-rank test will be used to compare weekly

attrition rates between treatment groups through month 9. We will also model weekly attrition rates using discrete-time Cox regression, with missed-visit indicators and with interim HbA1C and medication (type, dose) measures as time-varying predictors, and with treatment, age, gender, body habitus, and clinical measures as fixed predictors. Treatment compliance will be compared between groups using the Mann-Whitney rank-sum test. For the primary outcome, the potential impact of dropout will be assessed by simple imputation of missing outcomes based on hypothetical models for the missing outcomes conditional on observed data (e.g., prediction or carry forward). However, we know that such procedures artificially decrease nominal p-values and standard errors. If important changes in treatment comparisons emerge after simple imputation strategies, we will use MCMC-based multiple imputation (simulated draws from the predictive distribution of missing outcomes given the observed data) to properly evaluate the significance of treatment comparisons.[98, 98] Missing secondary outcomes will be treated similarly.

Standardization of Procedures and Quality Control. We will specify all procedures for recruitment and screening, informed consent, flow of activities, all measurements, training procedures, data entry and management, training of personnel, and other related matters. Procedures to calibrate all equipment also have been specified. Staff will undergo rigorous training to standardize all procedures and will be certified. They will be required to review the MOP annually for re-certification.

Strengths and Limitations. The study has limitations because its sample is limited to individuals with T2D. Thus, we will not know if the results will apply to individuals with pre-diabetes or non-diabetic populations. Finally, we stress that the purpose of HART-D is to evaluate the effect of different modes of exercise training on HbA1C. It will be conducted in near ideal circumstances, with relatively motivated participants, well-trained personnel, extensive efforts (including a stipend) to ensure adherence, and a well-equipped exercise facility. Many important questions of interest to the public and to clinicians will be addressed, including: "Can RT alone reduce HbA1C? Is the AT+RT better at reducing HbA1C then either of them alone?" Information from HART-D may contribute to better understanding of the mechanisms responsible for the substantial health benefits associated with aerobic and/or resistance training in individuals with T2D and may lead to better exercise guidelines for this at risk population.

Reference List

(1) American Diabetes Association. Physical activity/exercise and diabetes mellitus. *Diabetes Care* 2003 January;26 Suppl 1:S73-S77.

(2) Albright A, Franz M, Hornsby G et al. American College of Sports Medicine position stand. Exercise and type 2 diabetes. *Med Sci Sports Exerc* 2000 July;32(7):1345-60.

(3) American Diabetes Association. Physical activity/exercise and diabetes mellitus. *Diabetes Care* 2003 January;26 Suppl 1:S73-S77.

(4) Albright A, Franz M, Hornsby G et al. American College of Sports Medicine position stand. Exercise and type 2 diabetes. *Med Sci Sports Exerc* 2000 July;32(7):1345-60.

(5) Albright A, Franz M, Hornsby G et al. American College of Sports Medicine position stand. Exercise and type 2 diabetes. *Med Sci Sports Exerc* 2000 July;32(7):1345-60.

(6) Centers for Disease Control and Prevention. *National diabetes fact sheet: general information and national estimates on diabetes in the United States, 2000*. Atlanta, GA: U.S. Department of Health and Human Services, Centers for Disease Control and Prevention; 2002.

(7) Hamdy O, Goodyear LJ, Horton ES. Diet and exercise in type 2 diabetes mellitus. *Endocrinol Metab Clin North Am* 2001 December;30(4):883-907.

(8) Kelley DE, Goodpaster BH. Effects of exercise on glucose homeostasis in Type 2 diabetes mellitus. *Med Sci Sports Exerc* 2001 June;33(6 Suppl):S495-S501.

(9) American Diabetes Association. Physical activity/exercise and diabetes mellitus. *Diabetes Care* 2003 January;26 Suppl 1:S73-S77.

(10) Albright A, Franz M, Hornsby G et al. American College of Sports Medicine position stand. Exercise and type 2 diabetes. *Med Sci Sports Exerc* 2000 July;32(7):1345-60.

(11) Nelson KM, Reiber G, Boyko EJ. Diet and exercise among adults with type 2 diabetes: findings from the third national health and nutrition examination survey (NHANES III). *Diabetes Care* 2002 October;25(10):1722-8.

(12) Albright A, Franz M, Hornsby G et al. American College of Sports Medicine position stand. Exercise and type 2 diabetes. *Med Sci Sports Exerc* 2000 July;32(7):1345-60.

(13) Khaw KT, Wareham N, Luben R et al. Glycated haemoglobin, diabetes, and mortality in men in Norfolk cohort of european prospective investigation of cancer and nutrition (EPIC-Norfolk). *BMJ* 2001 January 6;322(7277):15-8.

(14) UK Prospective Diabetes Study (UKPDS) Group. Intensive blood-glucose control with sulphonylureas or insulin compared with conventional treatment and risk of complications in patients with type 2 diabetes (UKPDS 33). *Lancet* 1998 September 12;352(9131):837-53.

(15) Boule NG, Haddad E, Kenny GP, Wells GA, Sigal RJ. Effects of exercise on glycemic control and body mass in type 2 diabetes mellitus: a meta-analysis of controlled clinical trials. *JAMA* 2001 September 12;286(10):1218-27.

(16) Ishii T, Yamakita T, Sato T, Tanaka S, Fujii S. Resistance training improves insulin sensitivity in NIDDM subjects without altering maximal oxygen uptake. *Diabetes Care* 1998 August;21(8):1353-5.

(17) Baldi JC, Snowling N. Resistance training improves glycaemic control in obese type 2 diabetic men. *Int J Sports Med* 2003 August;24(6):419-23.

(18) Castaneda C, Layne JE, Munoz-Orians L et al. A randomized controlled trial of resistance exercise training to improve glycemic control in older adults with type 2 diabetes. *Diabetes Care* 2002 December;25(12):2335-41.

(19) Dunstan DW, Daly RM, Owen N et al. High-intensity resistance training improves glycemic control in older patients with type 2 diabetes. *Diabetes Care* 2002 October;25(10):1729-36.

(20) Dunstan DW, Daly RM, Owen N et al. High-intensity resistance training improves glycemic control in older patients with type 2 diabetes. *Diabetes Care* 2002 October;25(10):1729-36.

(21) Castaneda C, Layne JE, Munoz-Orians L et al. A randomized controlled trial of resistance exercise training to improve glycemic control in older adults with type 2 diabetes. *Diabetes Care* 2002 December;25(12):2335-41.

(22) Castaneda C, Layne JE, Munoz-Orians L et al. A randomized controlled trial of resistance exercise training to improve glycemic control in older adults with type 2 diabetes. *Diabetes Care* 2002 December;25(12):2335-41.

(23) American Diabetes Association. Physical activity/exercise and diabetes mellitus. *Diabetes Care* 2003 January;26 Suppl 1:S73-S77.

(24) Albright A, Franz M, Hornsby G et al. American College of Sports Medicine position stand. Exercise and type 2 diabetes. *Med Sci Sports Exerc* 2000 July;32(7):1345-60.

(25) Kumar S, Boulton AJ, Beck-Nielsen H et al. Troglitazone, an insulin action enhancer, improves metabolic control in NIDDM patients. Troglitazone Study Group. *Diabetologia* 1996 June;39(6):701-9.

(26) Hanley AJ, Williams K, Stern MP, Haffner SM. Homeostasis model assessment of insulin resistance in relation to the incidence of cardiovascular disease: the San Antonio Heart Study. *Diabetes Care* 2002 July;25(7):1177-84.

(27) Katsuki A, Sumida Y, Gabazza EC et al. Homeostasis model assessment is a reliable indicator of insulin resistance during follow-up of patients with type 2 diabetes. *Diabetes Care* 2001 February;24(2):362-5.

(28) Hanley AJ, Williams K, Stern MP, Haffner SM. Homeostasis model assessment of insulin resistance in relation to the incidence of cardiovascular disease: the San Antonio Heart Study. *Diabetes Care* 2002 July;25(7):1177-84.

(29) Dumortier M, Brandou F, Perez-Martin A, Fedou C, Mercier J, Brun JF. Low intensity endurance exercise targeted for lipid oxidation improves body composition and insulin sensitivity in patients with the metabolic syndrome. *Diabetes Metab* 2003 November;29(5):509-18.

(30) Helge JW. Prolonged adaptation to fat-rich diet and training; effects on body fat stores and insulin resistance in man. *Int J Obes Relat Metab Disord* 2002 August;26(8):1118-24.

(31) Miyatake N, Takahashi K, Wada J et al. Daily exercise lowers blood pressure and reduces visceral adipose tissue areas in overweight Japanese men. *Diabetes Res Clin Pract* 2003 December;62(3):149-57.

(32) Stewart KJ. Exercise training and the cardiovascular consequences of type 2 diabetes and hypertension: plausible mechanisms for improving cardiovascular health. *JAMA* 2002 October 2;288(13):1622-31.

(33) Grossman E, Messerli FH, Goldbourt U. High blood pressure and diabetes mellitus: are all antihypertensive drugs created equal? *Arch Intern Med* 2000 September 11;160(16):2447-52.

(34) Kelley GA, Kelley KA, Tran ZV. Aerobic exercise and resting blood pressure: a meta-analytic review of randomized, controlled trials. *Prev Cardiol* 2001;4(2):73-80.

(35) Albright A, Franz M, Hornsby G et al. American College of Sports Medicine position stand. Exercise and type 2 diabetes. *Med Sci Sports Exerc* 2000 July;32(7):1345-60.

(36) Leon AS, Sanchez OA. Response of blood lipids to exercise training alone or combined with dietary intervention. *Med Sci Sports Exerc* 2001 June;33(6 Suppl):S502-S515.

(37) Leon AS. Effects of exercise conditioning on physiological precursors to coronary heart disease. *J Cardiopulm Rehab* 1991;11:46-57.

(38) Stefanick ML, Wood PD. Physical activity, lipid and lipoprotein metabolism, and lipid transport. In: Bouchard C, Shephard RJ, Stephens T, editors. *Physical Activity, Fitness, and Health*.Champaign, IL: Human Kinetics; 1994. p. 417-31.

(39) Taylor PA, Ward A. Women, high-density lipoprotein cholesterol, and exercise. *Arch Intern Med* 1993 May 24;153(10):1178-84.

(40) Durstine JL, Haskell WL. Effects of exercise training on plasma lipids and lipoproteins. *Exerc Sport Sci Rev* 1994;22:477-521.

(41) Maiorana A, O'Driscoll G, Goodman C, Taylor R, Green D. Combined aerobic and resistance exercise improves glycemic control and fitness in type 2 diabetes. *Diabetes Res Clin Pract* 2002 May;56(2):115-23.

(42) Ishii T, Yamakita T, Sato T, Tanaka S, Fujii S. Resistance training improves insulin sensitivity in NIDDM subjects without altering maximal oxygen uptake. *Diabetes Care* 1998 August;21(8):1353-5.

(43) Dunstan DW, Puddey IB, Beilin LJ, Burke V, Morton AR, Stanton KG. Effects of a short-term circuit weight training program on glycaemic control in NIDDM. *Diabetes Res Clin Pract* 1998 April;40(1):53-61.

(44) Castaneda C, Layne JE, Munoz-Orians L et al. A randomized controlled trial of resistance exercise training to improve glycemic control in older adults with type 2 diabetes. *Diabetes Care* 2002 December;25(12):2335-41.

(45) Dunstan DW, Daly RM, Owen N et al. High-intensity resistance training improves glycemic control in older patients with type 2 diabetes. *Diabetes Care* 2002 October;25(10):1729-36.

(46) Eriksson J, Taimela S, Eriksson K, Parviainen S, Peltonen J, Kujala U. Resistance training in the treatment of non-insulin-dependent diabetes mellitus. *Int J Sports Med* 1997 May;18(4):242-6.

(47) Honkola A, Forsen T, Eriksson J. Resistance training improves the metabolic profile in individuals with type 2 diabetes. *Acta Diabetol* 1997 December;34(4):245-8.

(48) Haffner SM. Insulin resistance, inflammation, and the prediabetic state. *Am J Cardiol* 2003 August 18;92(4A):18J-26J.

(49) Ridker PM, Hennekens CH, Buring JE, Rifai N. C-reactive protein and other markers of inflammation in the prediction of cardiovascular disease in women. *N Engl J Med* 2000 March 23;342(12):836-43.

(50) Ridker PM. High-sensitivity C-reactive protein: potential adjunct for global risk assessment in the primary prevention of cardiovascular disease. *Circulation* 2001 April 3;103(13):1813-8.

(51) Ridker PM, Rifai N, Clearfield M et al. Measurement of C-reactive protein for the targeting of statin therapy in the primary prevention of acute coronary events. *N Engl J Med* 2001 June 28;344(26):1959-65.

(52) Lagrand WK, Visser CA, Hermens WT et al. C-reactive protein as a cardiovascular risk factor: More than an epiphenomenon? *Circulation* 1999 July 6;100(1):96-102.

(53) Ridker PM. Clinical application of C-reactive protein for cardiovascular disease detection and prevention. *Circulation* 2003 January 28;107(3):363-9.

(54) Geffken DF, Cushman M, Burke GL, Polak JF, Sakkinen PA, Tracy RP. Association between Physical Activity and Markers of Inflammation in a Healthy Elderly Population. *Am J Epidemiol* 2001 February 1;153(3):242-50.

(55) Rohde LE, Hennekens CH, Ridker PM. Survey of C-reactive protein and cardiovascular risk factors in apparently healthy men. *Am J Cardiol* 1999 November 1;84(9):1018-22.

(56) Ford ES. Does exercise reduce inflammation? Physical activity and C-reactive protein among U.S. adults. *Epidemiology* 2002 September;13(5):561-8.

(57) LaMonte MJ, Durstine JL, Yanowitz FG et al. Cardiorespiratory fitness and C-reactive protein among a tri-ethnic sample of women. *Circulation* 2002 July 23;106(4):403-6.

(58) Tomaszewski M, Charchar FJ, Przybycin M et al. Strikingly Low Circulating CRP Concentrations in Ultramarathon Runners Independent of Markers of Adiposity: How Low Can You Go? *Arterioscler Thromb Vasc Biol* 2003 September 1;23(9):1640-4.

(59) Church TS, Barlow CE, Earnest CP, Kampert JB, Priest EL, Blair SN. Associations between cardiorespiratory fitness and C-reactive protein in men. *Arteriosclerosis Thrombosis Vascular Biology* 2002;22:1869-76.

(60) Nguyen-Duy TB, Nichaman MZ, Church TS, Blair SN, Ross R. Visceral fat and liver fat are independent predictors of metabolic risk factors in men. *Am J Physiol* 2003;284:E1065-E1072.

(61) Yarnell JW, Patterson CC, Thomas HF, Sweetnam PM. Central obesity: predictive value of skinfold measurements for subsequent ischaemic heart disease at 14 years follow-up in the Caerphilly Study. *Int J Obes Relat Metab Disord* 2001 October;25(10):1546-9.

(62) Irwin ML, Yasui Y, Ulrich CM et al. Effect of exercise on total and intra-abdominal body fat in postmenopausal women: a randomized controlled trial. *JAMA* 2003 January 15;289(3):323-30.

(63) Mourier A, Gautier JF, De Kerviler E et al. Mobilization of visceral adipose tissue related to the improvement in insulin sensitivity in response to physical training in NIDDM. Effects of branched-chain amino acid supplements. *Diabetes Care* 1997 March;20(3):385-91.

(64) Treuth MS, Hunter GR, Kekes-Szabo T, Weinsier RL, Goran MI, Berland L. Reduction in intra-abdominal adipose tissue after strength training in older women. *J Appl Physiol* 1995 April;78(4):1425-31.

(65) Boule NG, Haddad E, Kenny GP, Wells GA, Sigal RJ. Effects of exercise on glycemic control and body mass in type 2 diabetes mellitus: a meta-analysis of controlled clinical trials. *JAMA* 2001 September 12;286(10):1218-27.

(66) Castaneda C, Layne JE, Munoz-Orians L et al. A randomized controlled trial of resistance exercise training to improve glycemic control in older adults with type 2 diabetes. *Diabetes Care* 2002 December;25(12):2335-41.

(67) Dunstan DW, Daly RM, Owen N et al. High-intensity resistance training improves glycemic control in older patients with type 2 diabetes. *Diabetes Care* 2002 October;25(10):1729-36.

(68) American Diabetes Association. Physical activity/exercise and diabetes mellitus. *Diabetes Care* 2003 January;26 Suppl 1:S73-S77.

(69) Albright A, Franz M, Hornsby G et al. American College of Sports Medicine position stand. Exercise and type 2 diabetes. *Med Sci Sports Exerc* 2000 July;32(7):1345-60.

(70) Kohl HW, III, Dunn AL, Marcus BH, Blair SN. A randomized trial of physical activity interventions:design and baseline data from Project Active. *Med Sci Sports Exerc* 1998 February;30(2):275-83.

(71) Blair SN, Applegate WB, Dunn AL et al. Activity Counseling Trial (ACT): rationale, design, and methods. Activity Counseling Trial Research Group. *Med Sci Sports Exerc* 1998 July;30(7):1097-106.

(72) Schulz KF, Grimes DA. Allocation concealment in randomised trials: defending against deciphering. *Lancet JID - 2985213R* 2002 February 16;359(9306):614-8.

(73) Matthews DR, Hosker JP, Rudenski AS, Naylor BA, Treacher DF, Turner RC. Homeostasis model assessment: insulin resistance and beta-cell function from fasting plasma glucose and insulin concentrations in man. *Diabetologia* 1985 July;28(7):412-9.

(74) Castaneda C, Layne JE, Munoz-Orians L et al. A randomized controlled trial of resistance exercise training to improve glycemic control in older adults with type 2 diabetes. *Diabetes Care* 2002 December;25(12):2335-41.

(75) Haskell WL. What to look for in assessing responsiveness to exercise in a health context. *Med Sci Sports Exerc* 2001 June;33(6 Suppl):S454-S458.

(76) Kelley DE, Goodpaster BH. Effects of exercise on glucose homeostasis in Type 2 diabetes mellitus. *Med Sci Sports Exerc* 2001 June;33(6 Suppl):S495-S501.

(77) Smutok MA, Reece C, Kokkinos PF et al. Aerobic versus strength training for risk factor intervention in middle-aged men at high risk for coronary heart disease. *Metabolism* 1993;42(2):177-84.

(78) Smutok MA, Reece C, Kokkinos PF et al. Effects of exercise training modality on glucose tolerance in men with abnormal glucose regulation. *Int J Sports Med* 1994 August;15(6):283-9.

(79) Rice B, Janssen I, Hudson R, Ross R. Effects of aerobic or resistance exercise and/or diet on glucose tolerance and plasma insulin levels in obese men. *Diabetes Care* 1999 May;22(5):684-91.

(80) American Diabetes Association. Physical activity/exercise and diabetes mellitus. *Diabetes Care* 2003 January;26 Suppl 1:S73-S77.

(81) Albright A, Franz M, Hornsby G et al. American College of Sports Medicine position stand. Exercise and type 2 diabetes. *Med Sci Sports Exerc* 2000 July;32(7):1345-60.

(82) NIH Consensus Development Panel on Physical Activity and Cardiovascular Health. Physical activity and cardiovascular health. *JAMA* 1996;276(3):241-6.

(83) Pate RR, Pratt M, Blair SN et al. Physical activity and public health: a recommendation from the Centers for Disease Control and Prevention and the American College of Sports Medicine. *JAMA* 1995;273:402-7.

(84) Hagberg JM, Montain SJ, Martin WH, Ehsani AA. Effect of exercise training in 60- to 69-year-old persons with essential hypertension. *Am J Cardiol* 1989;64(5):348-53.

(85) U.S.Department of Health and Human Services. *Physical activity and health: A report of the Surgeon General.* Atlanta, GA: U.S. Department of Health and Human Services, Centers for Disease Control and Prevention, National Center for Chronic Disease Prevention and Health Promotion; 1996.

(86) Snyder KA, Donnelly JE, Jacobsen DJ, Hertner G, Jakicic JM. The effects of long-term, moderate intensity, intermittent exercise on aerobic capacity, body composition, blood lipids, insulin and glucose in overweight females. *Int J Obes Relat Metab Disord* 1997;21:1180-9.

(87) Ready AE, Drinkwater DT, Ducas J, Fitzpatrick DW, Brereton DG, Oades SC. Walking program reduces elevated cholesterol in women post-menopause. *Can J Cardiol* 1995 November;11(10):905-12.

(88) Leon AS. Contributions of regular moderate-intensity physical activity to reduced risk of coronary heart disease. In: Leon AS, editor. *National Institutes of Health, physical activity and cardiovascular health: A national consensus.*Champaign, IL: Human Kinetics; 1997. p. 57-66.

(89) American Diabetes Association. Physical activity/exercise and diabetes mellitus. *Diabetes Care* 2003 January;26 Suppl 1:S73-S77.

(90) Albright A, Franz M, Hornsby G et al. American College of Sports Medicine position stand. Exercise and type 2 diabetes. *Med Sci Sports Exerc* 2000 July;32(7):1345-60.

(91) Maiorana A, O'Driscoll G, Goodman C, Taylor R, Green D. Combined aerobic and resistance exercise improves glycemic control and fitness in type 2 diabetes. *Diabetes Res Clin Pract* 2002 May;56(2):115-23.

(92) Rhea MR, Alvar BA, Burkett LN, Ball SD. Meta-analysis to determine the dose response for strength development. *Med Sci Sports Exerc* 2003;35(3):456-64.

(93) Albright A, Franz M, Hornsby G et al. American College of Sports Medicine position stand. Exercise and type 2 diabetes. *Med Sci Sports Exerc* 2000 July;32(7):1345-60.

(94) Willey KA, Singh MA. Battling insulin resistance in elderly obese people with type 2 diabetes: bring on the heavy weights. *Diabetes Care* 2003 May;26(5):1580-8.

(95) Dishman RK. *Exercise adherence: Its impact on public health.* Champaign, IL: Human Kinetics; 1988.

(96) Martin JE, Dubbert PM. Exercise applications and promotion in behavioral medicine:current status and future directions. *J Consult Clin Psych* 1982;50:1004-17.

(97) O'Neal HA, Blair SN. Enhancing adherence in clinical exercise trials. *Quest* 2001;53:310-7.

(98) Little RJA, Rubin DB. *Statistical analysis with missing data.* New York, NY: John Wiley & Sons; 1987.
